# Pregnancy and Breastfeeding During COVID-19 Pandemic: A Systematic Review of Published Pregnancy Cases

**DOI:** 10.3389/fpubh.2020.558144

**Published:** 2020-11-23

**Authors:** Carina Rodrigues, Inês Baía, Rosa Domingues, Henrique Barros

**Affiliations:** ^1^EPIUnit – Instituto de Saúde Pública, Universidade do Porto, Porto, Portugal; ^2^Instituto Nacional de Infectologia Evandro Chagas, Fundação Oswaldo Cruz, Rio de Janeiro, Brazil

**Keywords:** COVID-19, pregnancy, vertical transmission, perinatal outcomes, breastfeeding

## Abstract

**Background:** The COVID-19 pandemic is an emerging concern regarding the potential adverse effects during pregnancy. This study reviews knowledge on the impact of COVID-19 on pregnancy and describes the outcome of published cases of pregnant women diagnosed with COVID-19.

**Methods:** Searches were conducted in PubMed®, Scopus®, Web of Science®, and MedRxiv® up to 26th June 2020, using PRISMA standards, to identify original published studies describing pregnant women at any gestational age diagnosed COVID-19. There were no date or language restrictions on the search. All identified studies were included irrespective of assumptions on study quality.

**Results:** We identified 161 original studies reporting 3,985 cases of pregnant women with COVID-19 (1,007 discharged while pregnant). The 2,059 published cases with pregnancy outcomes resulted in 42 abortions, 21 stillbirths, and 2,015 live births. Preterm birth occurred in 23% of cases. Around 6% of pregnant women required admission to an intensive care unit and 28 died. There were 10 neonatal deaths. From the 163 cases with amniotic fluid, placenta, and/or cord blood analyzed for the SARS-CoV-2 virus, 10 were positive. Sixty-one newborns were positive for SARS-CoV-2. Four breast milk samples from 92 cases showed evidence of SARS-CoV-2.

**Conclusion:** Emerging evidence suggests that vertical transmission is possible, however, there is still a limited number of reported cases with intrapartum samples. Information, counseling and adequate monitoring are essential to prevent and manage adverse effects of SARS-CoV-2 infection during pregnancy.

## Introduction

The disease resulting from infection with the Severe Acute Respiratory Syndrome Coronavirus 2 (SARS-COV-2) and designated COVID-19 by the World Health Organization (WHO) was first identified in humans in December 2019, in the city of Wuhan, China (1), and can present from asymptomatic to a severe acute respiratory infection requiring intensive care (2, 3). The infection can occur at any age, but COVID-19 is proportionally uncommon in children (<1% of the total cases). The infection fatality rate is around 1% but much higher in older people or those with pre-existing medical conditions (such as heart disease, diabetes, chronic obstructive pulmonary disease) (2, 4).

Person-to-person transmission of COVID-19 is well-established and can occur when an infected person coughs, sneezes, or speaks and scattered droplets are inhaled or reach the mucous membranes of the mouth, nose, or eyes of susceptible. COVID-19 can also be transmitted through direct hand contact with surfaces or objects contaminated with SARS-CoV-2 followed by contact with the mouth, nose, or eyes (2).

Pregnant women and newborns receive special attention and there is an emerging concern with the potential risk of SARS-COV-2 vertical transmission (from mother to fetus) or associated malformations, and contagion during delivery and breastfeeding; likewise, it is important to determine the potential adverse effects of COVID-19 in pregnant women (5–7). Considering the rapidly evolving of the COVID-19 pandemic, which is reflected in the current lack of high-quality evidence, we aimed to review the published cases of pregnant women diagnosed with COVID-19.

## Methods

The review follows the Preferred Reporting of Systematic Reviews and Meta-Analysis (PRISMA) guidelines (8, 9). This review was not registered with PROSPERO. We searched PubMed®, Scopus®, Web of Science®, and MedRxiv® electronic databases up to 26th June 2020 to identify original published studies describing pregnant women at any gestational age diagnosed with COVID-19 [confirmed by clinical/radiological evidence of pneumonia compatible with SARS-CoV-2 and/or by quantitative real-time polymerase chain reaction (PCR) or dual fluorescence PCR of SARS-CoV-2 infection]. The following search expression was used [(COVID-19 OR 2019-nCoV OR “novel coronavirus” OR SARS-CoV-2 OR “coronavirus 2”) AND (pregnancy OR delivery OR pregnant OR obstetric^*^ OR maternal OR perinatal OR breastfeeding)]. Also, reference tracking was carried out to identify other potential studies to be included.

The PECO (Population, Exposure, Comparison, Outcome) structure design was used to define exposure, outcome, as well as inclusion and exclusion criteria for the review (Table 1). The question was “What are the main obstetric, maternal, and neonatal outcomes of SARS-CoV-2 infection during pregnancy and the potential risk of vertical transmission?”

**Table 1:** PECO criteria for inclusion and exclusion of studies.

**Parameter**	**Inclusion criteria**	**Data extraction**
Population (P)	Pregnant women at any gestational age	Country, maternal age, gestational age at birth (or at admission), comorbidities
Exposure (E)	COVID-19 diagnose	SARS-CoV-2 infection
Comparison (C)	None	
Outcome (O)	Pregnancy, maternal, and neonatal outcomes	Pregnancy complications, type of delivery, indication for cesarean section, maternal admission to intensive care unit, maternal death, birth weight, neonatal complications, breastfeeding, intrauterine, and/or neonatal samples collected for detection of SARS-CoV-2

Each reference retrieved was screened independently by two researchers following predefined criteria to determine eligibility for the systematic review. Studies were excluded if: (1) did not involve humans (e.g., *in vitro* or animal research); (2) non-original articles (e.g., book chapters, review articles, guidelines); (3) data not reporting pregnant women diagnosed with COVID-19; (4) only indicate prevalence estimations among pregnant women, with no description of perinatal outcomes; and (5) reporting breastfeeding after puerperium period, with no information about pregnancy. There were no date or language restrictions on the search.

Two researchers independently reviewed the included studies and extracted the following data: type of study, data collection period, maternal age, comorbidities and pregnancy complications, type of delivery, indication for cesarean section, gestational age at birth (or at admission or diagnosis), pregnancy outcome, maternal admission to intensive care unit (including mechanical ventilation), maternal death, neonatal outcomes (birth weight, neonatal complications, neonatal death, breastfeeding), intrauterine and/or neonatal samples collected for detection of SARS-CoV-2 (such as amniotic fluid, umbilical cord blood, placenta, breast milk, nasopharyngeal, and anal swabs), and their results (negative/positive and/or reactive/non-reactive).

All identified original observational studies reporting cases of pregnant women at any gestational age diagnosed with COVID-19 were included irrespective of study quality (preprints preliminary reports were also included). The research design of the studies was described based on the authors' classification, except for case reports which were considered when the manuscript described only one case.

Doubts on possible duplicates and/or differences in the data extraction were discussed and resolved by consensus, involving a third researcher whenever necessary.

Cases reported in more than one study, and for which it was possible to identify duplicates, were described only once, presenting the more detailed data. We identified duplicates based on author names and hospital location, publication date, participant admission date, maternal and neonatal characteristics, and outcomes. We did not contact the corresponding authors because of the time constraints and the importance to have immediate results.

Considering the heterogeneity observed across the studies, we decided to perform a narrative synthesis using the Synthesis Without Meta-analysis (SWiM) reporting guideline (intended to complement the PRISMA guidelines) (10). Descriptive statistics were presented (frequency and proportions) based on the total cases with available information.

## Results

**Figure 1:**
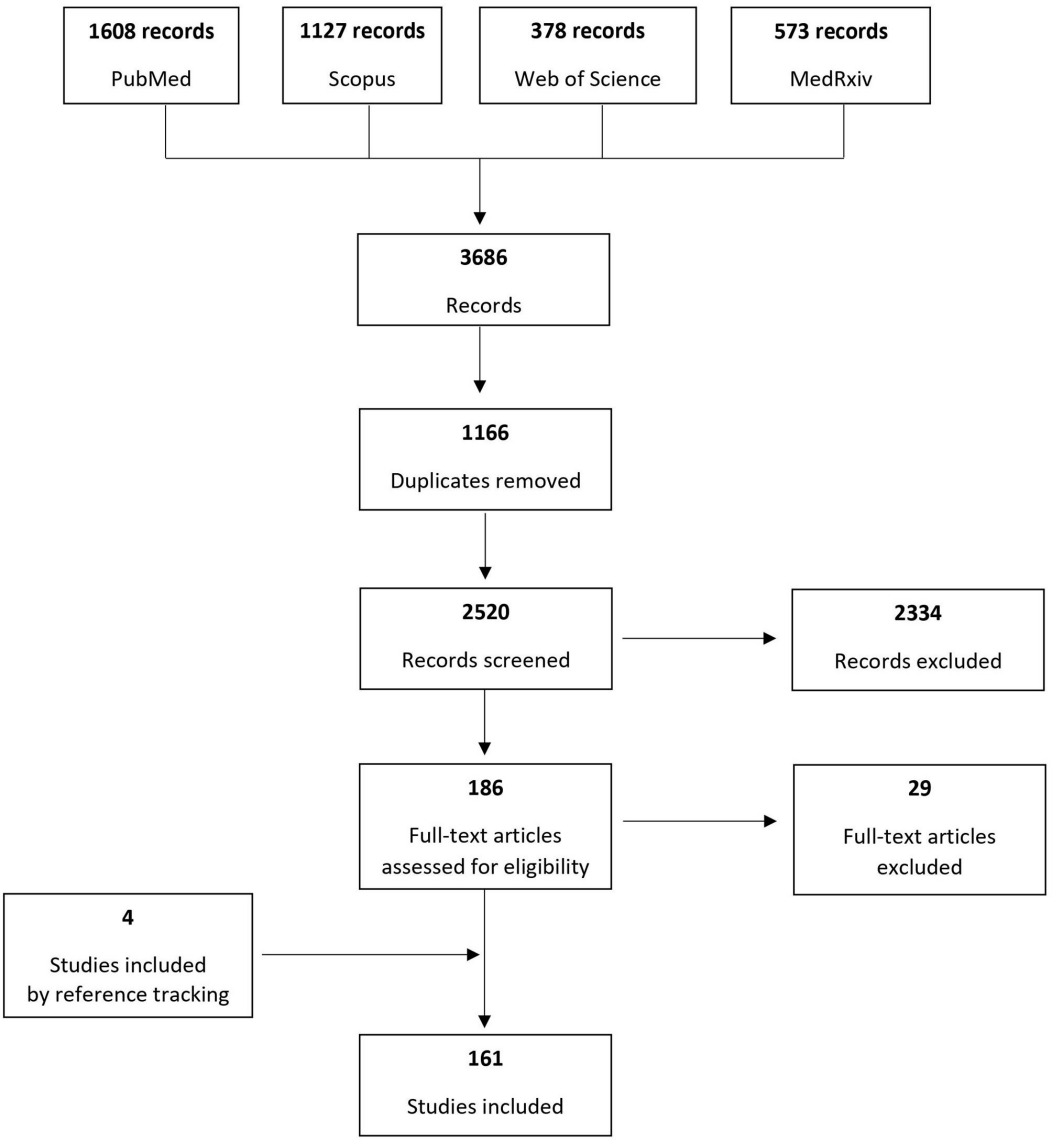
Systematic review flowchart.

Based on the search of the four electronic databases, 3,686 records were identified and after removing duplicates 2,520 were screened to assess their eligibility for inclusion. One hundred sixty-one original studies published until June 26th, 2020 were included, reporting cases of pregnant women at any gestational age diagnosed with COVID-19. Figure 1 presents the process of study inclusion in the systematic review.

From the 161 studies included, 66 were case series reports (48% from China and 23% from USA) (11–76), 59 case reports (27% from China and 25% from USA) (77–135), 20 cohort studies (30% from China, 30% from USA, 15% from Spain) (136–155), 13 cross-sectional (62% China and 15% USA) (156–168), and 3 case-control studies (2 from China and 1 from Italy) (169–171). Table 2 shows the distribution of studies according to the study design.

**Table 2:** Distribution of references included in the review by study design and country.

**Design^**a**^**	**Country**	**Number of reports (*n* = 161)**
**Case report (*****n*** **=** **59)**		
	Australia	1
	Belgium	1
	Canada	1
	China	16
	France	1
	Germany	1
	Honduras	1
	India	1
	Iran	2
	Italy	3
	Jordan	1
	Korea	1
	Netherlands	2
	Peru	1
	Portugal	2
	Spain	1
	Sweden	1
	Switzerland	1
	Thailand	1
	Turkey	2
	UK	3
	USA	15
**Case series reports (*****n*** **=** **66)**		
	Brazil	1
	Canada	1
	China	32
	France	2
	India	1
	Iran	1
	Italy	5
	Mexico	1
	Portugal	1
	Spain	3
	Turkey	1
	UK	2
	USA	15
**Case-control (*****n*** **=** **3)**		
	China	2
	Italy	1
**Cross-sectional (*****n*** **=** **13)**		
	China	8
	Italy	1
	Japan	1
	Sweden	1
	USA	2
**Cohort (*****n*** **=** **20)**		
	China	6
	Denmark	1
	Israel	1
	Italy	2
	Spain	3
	UK	1
	USA	6

a *The studies' design was reported according to the authors' classification, except for case reports which were considered only when the paper described 1 case*.

The studies reported 3,985 pregnant women diagnosed with COVID-19 and the main characteristics are summarized in the Table 3. A detailed description of reported cases is presented in Supplementary Table 1.

**Table 3:** Characteristics of pregnant women diagnosed with COVID-19 described in the literature (*n* = 3985).

**Characteristics**	**n/N (%)**
**Maternal characteristics**	
Age, years (min-max)	15–49
Country of hospital admission	
United States of America	1206/3985 (30.26)
China	823/3985 (20.65)
France	672/3985 (16.86)
UK	440/3985 (11.04)
Mexico	308/3985 (7.73)
Spain	241/3985 (6.05)
Italy	202/3985 (5.07)
Portugal	14/3985 (0.35)
Sweden	14/3985 (0.35)
Denmark	13/3985 (0.33)
Israel	11/3985 (0.28)
Iran	11/3985 (0.28)
Turkey	10/3985 (0.25)
India	3/3985 (0.08)
Brazil	2/3985 (0.05)
Canada	2/3985 (0.05)
Japan	2/3985 (0.05)
Netherlands	2/3985 (0.05)
Australia	1/3985 (0.03)
Belgium	1/3985 (0.03)
Germany	1/3985 (0.03)
Honduras	1/3985 (0.03)
South Korea	1/3985 (0.03)
Jordan	1/3985 (0.03)
Peru	1/3985 (0.03)
Switzerland	1/3985 (0.03)
Thailand	1/3985 (0.03)
**Pregnancy complications**	
Gestational diabetes	179/3985 (4.49)
PROM/PPROM	108/3985 (2.71)
Preeclampsia/Eclampsia/ HELLP Syndrome	68/3985 (1.71)
Fetal distress	44/3985 (1.10)
Gestational hypertension	23/3985 (0.58)
Fetal growth restriction	17/3985 (0.43)
Placenta previa/ placental abruption/ placenta accreta	15/3985 (0.38)
Oligohydramnios/Polyhydramnios	7/3985 (0.18)
**Chronic diseases of pregnant women**	
Obesity	530/3985 (13.30)
Asthma	117/3985 (2.94)
Chronic hypertension	73/3985 (1.83)
Diabetes mellitus	68/3985 (1.71)
Hypothyroidism	26/3985 (0.65)
**Pregnancy status**	
Still pregnant at discharge	1007/3985 (25.27)
Pregnancy outcome available (delivered and/or abortion)	2090/3985 (52.45)
No information about pregnancy status	888/3985 (22.28)
**Pregnancy outcomes (number of fetus/pregnant women)**	
No information	31/2090 (1.48)
Voluntary termination of pregnancy	16/2059 (0.78)
Medical termination of pregnancy	5/2059 (0.24)
Spontaneous abortion/fetal demise/ectopic pregnancy (<20 weeks of gestation)	42/2059 (2.04)
Stillbirth (≥20 weeks of gestation; including 1 twin pregnancy)	21/2059 (1.02)
Live birth (including 38 twin pregnancies and 1 triple)	2015/2059 (97.86)
Cesarean section (among delivered pregnant women)	1127/2090 (53.92)
Preterm birth (<37 weeks of gestation) (among live birth delivered)	455/1975 (23.04)
**Maternal outcomes**	
Maternal deaths	28/3803 (0.74)
Maternal admission to Intensive/Critical Care Unit	215/3365 (6.39)
Maternal mechanical ventilation (invasive and/or non-invasive)	160/2972 (5.38)
ECMO	5/2972 (0.50)
**Neonatal outcomes (live births**, ***n*** **=** **2015)**	
Neonatal deaths	10/1755 (0.57)
Any neonatal positive sample for SARS-CoV-2 confirmed by RT-PCR (newborns with at least one sample positive)	61/2015 (3.03)
High levels of SARS-CoV-2 IgM antibodies in neonatal blood	4/2015 (0.20)

The majority of cases occurred in the USA (*n* = 1,206, 30%), China (*n* = 823, 21%), France (*n* = 672, 17%), UK (*n* = 440, 11%), Mexico (*n* = 308, 8%), Spain (*n* = 241, 6%), and Italy (*n* = 202, 5%). Maternal age ranged from 15 to 49 years.

From the 3,985 pregnant women described, 2090 (52.4%) had a pregnancy outcome, 1007 (25.3%) were discharged during pregnancy (undelivered) and 888 (22.3%) had no information. From those with available information on gestational age (*n* = 1,896), 89% of women were in the third trimester of pregnancy (*n* = 1,685) and only 5% in the first trimester (*n* = 101).

### Vertical Transmission of COVID-19

Among the intrauterine samples analyzed, 11.9% of placentas (*n* = 8/67), 1.8% of amniotic fluid (*n* = 1/54), and 2.4% umbilical cord blood (*n* = 1/42) were positive for SARS-CoV-2 virus (Table 4).

**Table 4:** Type of intrauterine/ neonatal samples collected and results.

**Type of samples**	n/N^*^ (%)
**Intrauterine samples**	
Placenta	67
Positive results	8 (11.94)
Amniotic fluid	54
Positive results	1 (1.85)
Umbilical cord blood	42
Positive results	1 (2.38)
Measurement of SARS-CoV-2 IgM and IgG antibody in umbilical cord blood	2
Reactive IgM	0 (0.0)
Reactive IgG	1 (50.0)
**Neonatal samples**	
RT-PCR newborn's samples (not specified)	648
Positive results	19 (2.93)
RT-PCR newborn's oral swabs	1045
Positive results	36 (3.44)
RT-PCR newborn's anal swabs	38
Positive results	5 (13.16)
RT-PCR newborn's blood	20
Positive results	1 (5.00)
Measurement of SARS-CoV-2 IgM and IgG antibody in newborn's blood	71
Reactive IgM	4 (5.63)
Reactive IgG	7 (9.86)
RT-PCR breast milk	92
Positive results	4 (4.35)
Measurement of SARS-CoV-2 IgM and IgG antibody in breast milk	7
Reactive IgM	5 (71.43)
Reactive IgG	0 (0.0)

* *Newborns with at least one sample tested*.

We identified 61 (3.0%) newborns with at least one positive sample for SARS-CoV-2 confirmed by real-time reverse transcription-polymerase chain reaction (RT-PCR) (Table 3). Most studies detected the SARS-CoV-2 RNA by RT-PCR using samples from the newborn's nasopharyngeal or throat (*n* = 1,045), sample collection varying from immediately to 17 days after birth. Neonatal serum samples were tested in 71 newborns, being four reactive for IgM and seven for IgG (Table 4).

From the 92 newborns with at least one breast milk sample tested (sample collection varying from immediately after birth to 15 days after), 4 were positive for SARS-CoV-2 virus by RT-PCR and 5 reactive for IgM antibodies (Table 4). One study reported 3 out of 5 positive breast milk samples collected during the first 5 days after birth, neonatal nasopharyngeal samples were negative on day 1 and 5 and the newborn received expressed breast milk (15).

### Maternal and Neonatal Outcomes

The obstetric conditions most frequently reported were gestational diabetes (4.5%), premature rupture of membranes (PROM/PPROM) (2.7%), pre-eclampsia/eclampsia/HELLP syndrome (1.7%), fetal distress (1.1%), gestational hypertension (0.6%), fetal growth restriction (0.4%), placenta previa/placental abruption/ placenta accreta (0.4%), and oligohydramnios/polyhydramnios (0.2%) (Table 3). The included studies reported 13.3% of obesity among pregnant women with COVID-19, 2.9% of asthma, 1.8% of chronic hypertension, and 1.7% of diabetes mellitus (Table 3).

Two hundred and fifteen pregnant women required admission to an intensive care unit (6.4%), 5.4% were mechanically ventilated (*n* = 160), and 0.5% required ECMO (*n* = 15). Twenty-eight maternal deaths with COVID-19 were reported (0.7%).

Among 2,059 pregnant women with pregnancy outcomes available, 16 (0.8%) resulted in termination of pregnancy due to maternal concerns regarding COVID-19 and 5 (0.2%) by medical reasons; 42 (2.0%) were spontaneous abortions/fetal demise (<20 weeks of gestation), 21 (1.0%) stillbirths (≥20 weeks of gestation, including one twin pregnancy); and 2015 (97.9%) live births (including 38 twin pregnancies and one triple pregnancy). Cesarean section was the most common type of delivery: 53.9% among 2,090 delivered pregnant women. Preterm birth occurred in 23.0% (455/1975) among live birth delivered and with available information on gestational age. Most of preterm births were iatrogenic for maternal and/or fetal compromise. There were 10 neonatal deaths (0.6%).

Although most of breast milk samples from COVID-19 infected mothers have tested negative for the SARS-CoV-2 virus, most infants did not receive any breast milk, considering those with available information (59.9%, *n* = 227/379).

## Discussion

Worldwide, the incidence of infection in pregnant women at any gestational age is still unclear, as universal screening tests are not generally used, except in the presence of symptoms or at admission for delivery. In a New York's hospital that implemented SARS-CoV-2 testing in all pregnant women admitted for delivery, 15.4% of them were positive for SARS-CoV-2, but 87.9% were asymptomatic (172).

The clinical characteristics of COVID-19 were similar to those described in non-pregnant women, presenting mild or moderate symptoms (11, 19, 21, 32, 76, 160). A systematic review summarized the clinical manifestations of 108 pregnant women with confirmed COVID-19 and most of them presented fever (68%) and coughing (34%), and lymphocytopenia (59%) with elevated C-reactive protein (70%) (173).

Furthermore, pregnant women do not appear to be at increased risk of severe illness of SARS-CoV-2 infection compared with non-pregnant women in the general population (6, 145, 174). However, a study from Sweden suggests that the risk of being admitted to an intensive care unit may be higher in pregnant and postpartum women (*n* = 13) compared with non-pregnant women of similar age (*n* = 40) (156). Recently, the Centers for Disease Control and Prevention (CDC) COVID-19 surveillance indicates an increased risk of intensive care unit admissions (1 in 68 of pregnant vs. 1 in 110 non-pregnant women, crude risk ratio 1.6, 95% CI 1.3–1.9) and mechanical ventilation (1 in 195 of pregnant vs. 1 in 370 non-pregnant women, crude risk ratio 1.9, 95% CI 1.4–2.6), but no increase was observed in the rate of mortality (1 in 513 of pregnant vs. 1 in 400 of non-pregnant women, crude risk ratio 0.8, 95% CI 0.5–1.3) (175). However, a more severe presentation of COVID-19 is commonly described in pregnant women with chronic conditions, such as obesity, asthma, and diabetes (174).

Although most of the published cases confirm the absence of transmission of the SARS-CoV-2 virus antenatally or intrapartum, emerging evidence suggests that vertical transmission is possible (28, 30, 75, 77, 82, 151). However, this is still controversial due to a reduced number of reported cases with intrapartum samples (placenta, amniotic fluid, umbilical cord blood), large variability in the type of biological material analyzed and the time of its collection. Results from our review reveal that only 7.8% of women with pregnancy outcome had at least one intrauterine sample analyzed (*n* = 163/2090). If vertical transmission occurred in all the positive cases reported, the proportion of neonatal infection would be around 6.1% (*n* = 10/163). It is important to highlight that only two studies (one from Italy and another from Canada) reported three cases with positive RT-PCR for SARS-CoV-2 simultaneously on placenta and neonatal nasopharyngeal swabs samples (51, 97). Two of these neonates had positive samples collected at birth. Additionally, one termination of pregnancy and one miscarriage presented positive placental samples, without evidence of fetal SARS-CoV-2 infection (106, 121). Two studies also used maternal and neonatal serum samples to test for immunoglobulins M (IgM) and G (IgG) antibodies (30, 77). In a series of six cases that had blood collected after delivery evaluated, two of the newborns had high levels of IgG and IgM antibodies (>10 AU/mL) and three had high values of IgG antibodies with normal levels of IgM, but in none SARS-CoV-2 virus was detected by RT-PCR in the neonatal oropharyngeal exudate (30). A case study also reported high values of IgM and IgG antibodies in the newborn's blood at days 1 and 15 after delivery, but with RT-PCR for SARS-CoV-2 negative in five samples of nasopharyngeal exudates collected between the first 2 h and the 16th day of life (77).

Regarding the effect of the SARS-CoV-2 virus on the fetus, no congenital malformation has been reported so far associated to COVID-19. Higher risk of preterm birth has been reported as we observed 23% of preterm birth in our review compared to the estimated global preterm birth rate that is around 10–15% (176). In one study that compared groups of pregnant women with and without COVID-19, there were no significant differences in the occurrence of gestational diabetes, severe pre-eclampsia, PROM, fetal distress, meconium-stained amniotic fluid, premature delivery, neonatal asphyxia, and procedures for severe post-partum bleeding (31).

The maternal and neonatal outcomes observed so far are quite different from the two most serious coronavirus-related previous epidemics (6, 177–190). The first also appeared in China, in 2002-03, and was characterized by severe respiratory infections caused by the Severe Acute Respiratory Syndrome-Coronavirus (SARS-CoV) . The second occurred in 2012, initially in the Middle East, the Middle East Respiratory Syndrome—Coronavirus (MERS-CoV) (6, 177). These epidemics have demonstrated the ability of coronavirus to cause serious complications during pregnancy (179, 190), with worse prognosis in pregnant women than non-pregnant women (181, 191).

In the 2002 epidemic, 12 pregnant women were infected with SARS-CoV, with a fatality rate of 25% (190). Among the seven pregnant women infected in the first trimester, four had a miscarriage (190). Two of the five pregnant women infected during the second or third trimester had fetal growth restriction and four had a preterm delivery (one spontaneous; three induced by the maternal condition) (190). In a review of the pregnancy outcomes of 11 women infected with MERS-CoV, seven pregnant women required admission to the intensive care unit and three died, of which only one had one comorbidity (asthma). Two fetal deaths occurred, and three of nine newborns were preterm (179).

However, considering that SARS-CoV-2 has genetic homology and some clinical similarities to SARS-CoV and MERS-CoV, and the immunological and physiological changes that occur during pregnancy, such as in cell-mediated immunity or lung function, that affect both the susceptibility and the clinical severity of pneumonia, it is important to pay particular attention to the monitoring of pregnant women with COVID-19, because maternal and perinatal adverse outcomes are potentially relevant (6, 177). One study reported two asymptomatic pregnant women at admission for delivery that rapidly evolved to severe COVID-19 disease requiring admission to an intensive care unit (14). Also, we identified 28 maternal deaths, with an infection fatality rate less than 1%, being similar to the other general populations (4). One article from Iran reported seven maternal deaths among nine pregnant women with severe COVID-19, of which three resulted in four stillbirths (1 twin pregnancy) and one in two neonatal deaths (1 twin pregnancy) (41). After concluding our search on the electronic databases, a paper from Brazil reporting 124 maternal deaths of pregnant or postpartum women was published, describing 22.6% admissions to the intensive care unit, of whom 64% had invasive ventilation (192). This raises awareness for questions related to access to healthcare services which may impact on the natural history of disease and reflect worldwide disparities on maternal outcomes.

Thus, it is essential to prevent the infection of COVID-19 and any other viral respiratory infection, as these infections represent an increased risk for the pregnant woman and for the pregnancy itself (6, 193, 194). It is therefore extremely important that pregnant women adopt preventive actions for COVID-19 with great intensity (174). For managing suspected or confirmed SARS-CoV-2 infection in pregnant women, recommendations for health professionals and services have already been published (174, 193, 195–197).

Most women in this review had a cesarean section, many of them without a clear medical indication. The decision on the type of delivery in pregnant women with suspected or confirmed infection with COVID-19 should consider maternal and fetal clinical characteristics, as in normal practice, and not the diagnosis of COVID-19 infection *per se*. Thus, there is no obstetric contraindication to any mode of delivery, unless the pregnant woman's clinical condition implies an emergent decision (174).

Regarding breast milk samples, the reviewed studies described four cases with evidence of SARS-CoV-2, from the 92 reported (15, 74, 97, 148). Despite that, there is not enough scientific evidence to unequivocally state that there is possibility that mothers with COVID-19 can transmit the virus through breast milk. Therefore, recommendations should be based on the available data and the analogy with past circumstances and predictable costs and benefits. Breastfeeding is recognized as the best form of child feeding due to the countless benefits for both the mother and the newborn, including the protection against gastrointestinal and respiratory infections (198). Thus, considering the benefits of breastfeeding and the fact that the transmission of other respiratory viruses is insignificant through breast milk, there is no indication to stop breastfeeding. According to the recommendations of WHO/UNICEF (199) and the Center for Disease Control and Prevention (CDC) of the United States (193), women with suspected or confirmed infection with COVID-19 can initiate or continue breastfeeding as long as clinical conditions permit. The CDC indicates that the decision to initiate or continue breastfeeding must be determined by the mother with COVID-19, together with family members and health professionals (193).

The major strength of this review is the inclusion of all study designs, including case reports and case series. To the best of our knowledge, this is the first review presenting a detailed description of clinical outcomes of each case identified (Supplementary Table 1). Limitations of this systematic review should be acknowledged. Considerable heterogeneity was observed across the studies, which did not allow us to conduct a meta-analysis. On the other hand, we cannot guarantee that we were able to identify all the cases of pregnant women described in the literature. Possibly there were additional cases currently presented in other types of publications, such as reports. Also, considering the importance of summarizing all existing cases, we did not assess the quality of the studies included in this review. Several studies had missing outcome data and selective reporting bias could not be excluded. Additionally, there may be some cases which could be duplicated, namely the studies which did not describe clinical characteristics case by case.

## Conclusion

According to this review, preterm delivery seems to be more frequent among pregnant women with COVID-19. There is emerging evidence on possible vertical transmission (three positive results simultaneously in the placental samples and neonatal oral swabs for SARS-CoV-2 were reported), but the clinical relevance of the fetal infection is unclear. So far, there is not enough scientific evidence to unequivocally state that there is possibility that mothers with COVID-19 can transmit the virus through breast milk.

The maternal fatality rate was below 1% among the reported cases and hospitalizations in intensive care were less than 7%. Although the complications appear to be similar to those of non-pregnant women, services must be prepared to attend to complications, especially in pregnant women with comorbidities. Information, counseling and adequate monitoring are essential to prevent and manage adverse effects of SARS-CoV-2 infection during pregnancy.

## Data Availability Statement

The original contributions presented in the study are included in the article/Supplementary Materials, further inquiries can be directed to the corresponding author/s.

## Author Contributions

CR and HB: conceptualization, methodology, data curation, and original draft preparation. IB and RD: methodology, data curation, writing-reviewing, and editing. All authors read and approved the final manuscript.

## Conflict of Interest

The authors declare that the research was conducted in the absence of any commercial or financial relationships that could be construed as a potential conflict of interest.
