# Supplementary material for: Pregnancy and Breastfeeding During COVID-19 Pandemic: A Systematic Review of Published Pregnancy Cases
Source: Front Public Health. 2020 Nov 23;8:558144. doi: 10.3389/fpubh.2020.558144 (PMC7719788; doi:10.3389/fpubh.2020.558144)
Supplement: Supplementary file 1 [file Data_Sheet_1.PDF]

SUPPLEMENTAL MATERIAL

Table S1. Description of cases of pregnant women with a diagnose of COVID-19.

Reference	Data collection period/ Country	Case	Maternal age	Pregnancy complication and/or comorbidities	Type of delivery	C-section indication	Gestational age at birth	Pregnancy outcome	Admission to Intensive/Critical Care Unit	Maternal death	Birth Weight (g)	Neonatal complications	Intrauterine/ Neonatal Samples collected	Intrauterine/ Neonatal SARS-CoV-2 infection	Neonatal death
Ahmed et al. (88)	8 Apr 2020 UK	1 case	29	Obesity; type 2 diabetes mellitus; renal tubular acidosis; asthma; vitamin D deficiency; Polyhydramnios; COVID-19 pneumonia; basilar artery thrombosis	Caesarean	Clinical condition (worsened respiratory function)	31^{+0}	Live birth	Yes (intubated, mechanical ventilation)	Yes	NI	NICU admission	RT-PCR in neonatal sample (not specified)	Negative	No
Algarroba et al. (89)	Published on 13 May 2020 USA	1 case	40	Sepsis; pneumonia	Caesarean	Clinical condition (worsened respiratory function and metabolic acidosis)	29^{+0}	Live birth	NI (intubated, sedated, and started on a norepinephrine infusion)	No	1340	NI	RT-PCR neonatal nasopharyngeal swab collected on days 2 and 3 of life; placenta	Negative (neonatal swabs) Visualization of the SARS-CoV-2 virus in the placenta by electron microscopy	No
Alzamora et al. (90)	29 Mar-3 Apr 2020 Peru	1 case	41	Class II obesity; diabetes mellitus	Caesarean	Clinical condition (maternal respiratory	33^{+0}	Live birth	NI (intubated and placed on	No	2970	NICU admission; required respiratory support for	RT-PCR neonatal nasopharyngeal swab, 16	Positive RT-PCR	No

						compromised status)			mechanical ventilation)			12hours. At the sixth day of life, presented mild respiratory difficulty and sporadic cough requiring supplemental oxygen with nasal cannula. Normal imaging and laboratory testing.	and 48 hours after delivery.<br><br>IgM and IgG at birth and at days 4 and 5 after birth.	(16 and 48h after birth)<br><br>Non-reactive (neonatal IgM and IgG at birth and at days 4 and 5 after birth)	
Amorim et al. (33)	NI<br><br>Brazil	2 cases	NI	NI	Caesarean (n=2)	Elective	NI	NI	NI	Yes (n=2)	NI	NI	NI	NI	NI
Anderson J et al (116)	Published on May 2020<br><br>USA	1 case	35	Type 2 diabetes mellitus, asthma, class III obesity, COVID-19 pneumonia, ARDS	Pregnant	NA	GA at admission 22^{+2}	NA	Yes<br><br>(mechanical ventilation)	No	NA	NA	NA	NA	NA
Baergen et al. (12)	Published on May 2020<br><br>USA	1 case	35	Focal placenta accreta	Vaginal	NA	39^{+6}	Live birth	No	No	3650	No	RT-PCR in neonatal sample (not specified)	Negative	No
Baergen et al. (12)	Published on May 2020<br><br>USA	1 case	30	Group B Streptococcus carrier status	Vaginal	NA	38^{+0}	Live birth	No	No	3360	No	RT-PCR in neonatal sample (not specified)	Negative	No
Baergen et al. (12)	Published on May 2020	1 case	29	None	Vaginal	NA	40^{+4}	Live birth	No	No	3400	No	RT-PCR in neonatal sample	Negative	No

	USA												(not specified)		
Baergen et al. (12)	Published on May 2020 USA	1 case	40	None	Caesarean	NI	39^{+4}	Live birth	No	No	3720	No	RT-PCR in neonatal sample (not specified)	Negative	No
Baergen et al. (12)	Published on May 2020 USA	1 case	26	None	Vaginal	NA	39^{+2}	Live birth	No	No	3050	No	RT-PCR in neonatal sample (not specified)	Negative	No
Baergen et al. (12)	Published on May 2020 USA	1 case	40	Meconium, SGA	Vaginal	NA	37^{+0}	Live birth	No	No	2072	No	RT-PCR in neonatal sample (not specified)	Negative	No
Baergen et al. (12)	Published on May 2020 USA	1 case	19	Pneumonia, acute hypoxia	Vaginal	NA	38^{+0}	Live birth	No	No	2390	No	RT-PCR in neonatal sample (not specified)	Negative	No
Baergen et al. (12)	Published on May 2020 USA	1 case	28	Sickle cell trait	Vaginal	NA	40^{+3}	Live birth	No	No	3820	No	RT-PCR in neonatal sample (not specified)	Negative	No
Baergen et al. (12)	Published on May 2020 USA	1 case	37	Nuchal cord, Idiopathic thrombocytopenic purpura	Caesarean	NI	39^{+0}	Live birth	No	No	2415	No	RT-PCR in neonatal sample (not specified)	Negative	No

Baergen et al. (12)	Published on May 2020 USA	1 case	26	None	Vaginal	NA	40^{+1}	Live birth	No	No	3799	No	RT-PCR in neonatal sample (not specified)	Negative	No
Baergen et al. (12)	Published on May 2020 USA	1 case	40	Placenta previa; chronic diabetes	Caesarean	NI	36^{+0}	Live birth	No	No	2680	No	RT-PCR in neonatal sample (not specified)	Negative	No
Baergen et al. (12)	Published on May 2020 USA	1 case	38	None	Vaginal	NA	39^{+0}	Live birth	No	No	NI	No	RT-PCR in neonatal sample (not specified)	Negative	No
Baergen et al. (12)	Published on May 2020 USA	1 case	28	Hypertension	Vaginal	NA	40^{+0}	Live birth	No	No	3800	No	RT-PCR in neonatal sample (not specified)	Negative	No
Baergen et al. (12)	Published on May 2020 USA	1 case	40	Severe preeclampsia	Caesarean	NI	33^{+2}	Live birth	No	No	NI	No	RT-PCR in neonatal sample (not specified)	Negative	No
Baergen et al. (12)	Published on May 2020 USA	1 case	41	Group B Strep screen positive	Vaginal	NA	40^{+0}	Live birth	No	No	4115	No	RT-PCR in neonatal sample (not specified)	Negative	No
Baergen et al. (12)	Published on May 2020 USA	1 case	16	None	Vaginal	NA	32^{+2}	Live birth	No	No	3314	No	RT-PCR in neonatal sample (not specified)	Negative	No

Baergen et al. (12)	Published on May 2020<br><br>USA	1 case	36	Severe preeclampsia	Caesarean	NI	35^{+0}	Live birth (n=2, twin pregnancy)	No	No	NI	No	RT-PCR in neonatal sample (not specified)	Negative	No
Baergen et al. (12)	Published on May 2020<br><br>USA	1 case	23	None	Vaginal	NA	39^{+5}	Live birth	No	No	3580	No	RT-PCR in neonatal sample (not specified)	Negative	No
Baergen et al. (12)	Published on May 2020<br><br>USA	1 case	25	Group B Strep screen positive	Vaginal	NA	38^{+4}	Live birth	No	No	3920	No	RT-PCR in neonatal sample (not specified)	Negative	No
Baergen et al. (12)	Published on May 2020<br><br>USA	1 case	32	Hypothyroidism	Vaginal	NA	37^{+6}	Live birth	No	No	3160	No	RT-PCR in neonatal sample (not specified)	Negative	No
Bani et al. (91)	28 Mar Apr 2020<br><br>Jordan	1 case	29	None	Caesarean	Mother´s choice	39^{+3}	Live birth	No	No	2600	NI	Neonatal oral swab	Negative	No
Baud et al. (106)	20 Mar 2020<br><br>Switzerland	1 case	28	Obesity	Vaginal miscarriage (Severe uterine contraction, active fetal movements; fetal tachycardia)	NA	19^{+2}	Miscarriage	No	No	NA	NA	Placenta; amniotic fluid; vaginal swabs; swabs from the axillae, mouth, meconium, and fetal blood;	Positive (only placenta)<br><br>Negative (other samples)	NA

													fetal autopsy		
Blauvelt et al. (92)	Published on 8 May 2020<br><br>USA	1 case	34	Moderate asthma, gestational diabetes mellitus, class II obesity	Caesarean	Clinical condition (worsened respiratory function)	28^{+6}	Live birth	Yes<br><br>(mechanical ventilation)	No	1880	Resuscitated by the neonatal team and intubated for respiratory distress; leukopenia; neutropenia; lymphopenia; mild acidosis; Within hours of birth, the neonate was extubated and placed on continuous positive airway pressure ventilation	Neonatal oral and nasopharyngeal swabs (at 24 hours, 48 hours, and 14 days) and rectal swab (at 48 hours). IgM and IgG on day 5.	Negative (PCR negative and IgM and IgG non-reactive)	No
Blitz et al. (35)<br><br>Blitz et al. (152)	1 Mar – 6 May, 2020<br><br>USA	449 cases	NI	NI	NI	NI	NI	NI	No	No	NI	NI	NI	NI	NI
Blitz et al. (35)	1 Mar – 6 May 2020<br><br>USA	1 case	NI	NI	Pregnant	NA	GA at admission 3rd trimester	NA	Yes (without mechanical ventilation)	No	NA	NA	NA	NA	NA
Blitz et al. (35)	1 Mar – 6 May, 2020<br><br>USA	1 case	NI	NI	NI	NI	NI	Live birth	Yes (mechanical ventilation)	No	NI	NI	Neonatal nasopharyngeal swab (at 1st day of life)	Negative	No
Blitz et al. (35)	2 Mar – 9 Apr 2020	8 cases	Range: 15-49	Obesity (n=1); obstructive sleep apnea (n=1)	NI	NI	Live births: NI	Live birth (n=7)	Yes (n=8)	Yes (n=1)	NI	NI	NI	NI	NI

Blitz et al. (152)	USA						Stillbirth: 2nd trimester	Stillbirth (n=1)							
Breslin et al. (14)<br><br>Breslin et al. (13)	Mar 2020<br><br>USA	1 case	38	Diabetes type 2; intrahepatic	Caesarean	Arrest of descent	37^{+0}	Live birth	Yes (endotracheal intubation)	No	NI	None	RT-PCR in neonatal sample (not specified) at day 1	Negative	No
Breslin et al. (14)<br><br>Breslin et al. (13)	Mar 2020<br><br>USA	1 case	33	Diabetes type 2; chronic hypertension; asthma	Caesarean	Failed induction	37^{+5}	Live birth	Yes	No	NI	None	RT-PCR in neonatal sample (not specified) at day 1	Negative	No
Breslin et al. (14)<br><br>Breslin et al. (13)<br><br>Andrikopoulou et al. (34)	13-27 Mar 2020<br><br>USA	41 cases	Mean: 29.7	Asthma (n=7); diabetes type 2 (n=1); chronic hypertension (n=1); BMI ≥ 30 kg/m^2 (n=26)	Caesarean (n=6)<br><br>Vaginal (n=10)<br><br>Pregnant (n=25)	Non-reassuring fetal heart tones (n=3), repeat caesarean (n=2), arrest of descent (n=1), arrest of dilation (n=1), failed labor induction (n=1)	Mean: 37^{+0} (32^{+4}-38^{+6})	Live birth (n=16)	No	No	NI	NICU admission (n=3): prematurity at 34^{+6} weeks (n=1); evaluation of a congenitally diagnosed multicystic dysplastic kidney after delivery at 39^{+5} weeks (n=1); respiratory distress with concern for sepsis at 37^{+0} weeks (n=1)	RT-PCR in neonatal sample (not specified) (n=16)	Negative (n=16)	No
Browne et al. (117)	Published on 24 April 2020<br><br>USA	1 case	33	Asthma; migraine headaches	Pregnant (twin pregnancy)	NA	GA at diagnosis 23	NA	No	No	NA	NA	NA	NA	NA

Buonsenso et al. (15)	Mar 2020 Italy	1 case	42	None	Caesarean	NI	38^{+3}	Live birth	No	No	3390	No	RT-PCR neonatal nasopharyngeal swab on day 1, 3 and 15 of life. RT-PCR on placenta, umbilical cord blood and breast milk (day 11-14).	Negative Neonatal samples on day 1 and 3 negative. RT-PCR positive on day 15 (postnatal infection). Placenta, cord blood and breast milk negatives.	No
Buonsenso et al. (15) Buonsenso et al. (36)	Mar 2020 Italy	1 case	38	COVID-19 pneumonia	Caesarean	Fetal bradycardia	35^{+5}	Live birth	No	No	2300	No	RT-PCR neonatal nasopharyngeal swab on day 1 and 5 of life. RT-PCR on placenta, umbilical cord blood and breast milk.	Neonatal samples negative. RT-PCR test on the placenta and umbilical blood were positive. Breast milk tested positive on 3 out of 5 samples collected during the first 5 days after birth and tested negative when collected	No

														on day 14 to 17.	
Buonsenso et al._ (15)	Mar 2020<br><br>Italy	1 case	NI	NI	Spontaneous abortion	NI	8	Spontaneous abortion	NI	No	NA	NA	NA	NA	NA
Buonsenso et al. (15)	Mar 2020<br><br>Italy	1 case	NI	Preeclampsia	Pregnant	NA	35^{+0}	NA	No	No	NA	NA	NA	NA	NA
Buonsenso et al. (15)	Mar 2020<br><br>Italy	1 case	NI	NI	Pregnant	NA	20^{+0}	NA	No	No	NA	NA	NA	NA	NA
Buonsenso et al. (15)	Mar 2020<br><br>Italy	1 case	NI	NI	Pregnant	NA	27^{+0}	NA	No	No	NA	NA	NA	NA	NA
Buonsenso et al. (15)	Mar 2020<br><br>Italy	1 case	NI	NI	Pregnant	NA	18^{+0}	NA	No	No	NA	NA	NA	NA	NA
Buonsenso et al. (15)	Published April 26<br><br>Italy	1 case	31	None	Pregnant	NA	GA at admission 24 weeks	NA	Yes (Non-invasive ventilation)	No	NA	NA	NA	NA	NA
Buonsenso et al. (15)	Published April 26<br><br>Italy	1 case	39	None	Pregnant	NA	GA at admission 17 weeks	NA	No	No	NA	NA	NA	NA	NA
Buonsenso et al. (36)	Published April 26<br><br>Italy	1 case	42	None	Caesarean	No	40	Live birth	No	No	NI	None	RT-PCR in neonatal sample (not specified); cord blood	Negative	No
Campbell et al. (16)	2-29 Apr 2020<br><br>USA	30 cases	<30 (n=14)<br><br>30-34 (n=10)<br><br>≥35 (n=6)	NI	Caesarean (n=10)<br><br>Vaginal (n=20)	NI	Term (n=30)	Live birth (n=30)	NI	NI	Mean: 3370 ± 621	NI	RT-PCR in neonatal nasopharyngeal swabs (24h of age) (n=30)	Negative (n=30)	NI

Carosso et al. (118)	April 2020<br><br>Italy	1 case	28	Gestational diabetes	Vaginal	NA	37	Live birth	No	No	3120	NI<br><br>(NICU admission)	RT-PCR in neonatal nasopharyngeal swab (at birth and 37 hours after birth); vaginal, rectal, stool, colostrum maternal swabs; placenta swabs (maternal and fetal sides). IgM and IgG on cord blood.	Maternal rectal and stool swabs post-delivery: Positive. Vaginal swab, placental swabs and colostrum: negative. Neonatal swab positive at birth; Neonatal swab negative at 37 hours after birth.<br><br>Cord blood: IgG positive, IgM negative	No
Chen et al. (18)	8 Dec 2019-20 Mar 2020<br><br>China	118 cases	Median: 31 (IQR 28-34)	NI	Spontaneous abortions (n=3)<br><br>Ectopic pregnancies (n=2)<br><br>Induced abortions due to patients'	Obstetrical indication (n=24)<br><br>COVID-19 (n=38)	1st trimester (n=22)<br><br>2nd trimester (n=21)<br><br>3rd trimester (n=75)<br><br>Preterm births (n=14/68)	Live birth (n=70; 2 twin pregnancies)	NI<br>(One woman received non-invasive mechanical ventilation after birth)	No	NI	None	Neonatal throat swabs (n=8), breast milk (n=3)	Negative	No

					concerns about COVID-19 (n=4)<br><br>Caesarean (n=63)<br><br>Vaginal (n=5)<br><br>Pregnant (n=41)										
Chen et al. (22)	19 Jan-10 Feb 2020<br><br>China	3 cases	NI	Fetal distress	Voluntary termination of pregnancy (n=1)<br><br>Pregnant (n=1)<br><br>Caesarean (n=1)	Fetal distress	Voluntary termination of pregnancy: 1st trimester<br><br>Pregnant: 2nd trimester (n=1)<br><br>Caesarean: 3rd trimester (n=1)	Live birth (n=1)	NI	No	NI	NI	RT-PCR neonatal nasopharyngeal swab, amniotic fluid, breast milk and cord blood	Negative (n=1)	No
Chen et al. (20)	22 Jan - 25 Feb 2020<br><br>China	1 case	23	Placenta previa	Caesarean	Placenta previa	37^{+4}	Live birth	No	No	NI	None	Placenta; neonatal throat swab	Negative	No
Chen et al. (20)	22 Jan - 25 Feb 2020<br><br>China	1 case	34	Cholecystitis; placental abruption	Caesarean	Cholecystitis; placental abruption	39^{+0}	Live birth	No	No	NI	None	Placenta; neonatal throat swab	Negative	No

Chen et al. (20)	22 Jan - 25 Feb 2020<br><br>China	1 case	32	Placenta previa	Caesarean	Placenta previa	35^{+0}	Live birth	No	No	Low birth weight	None	Placenta; neonatal throat swab	Negative	No
Chen et al. (21)	20 Jan - 10 Feb 2020<br><br>China	1 case	29	Gestational diabetes	Vaginal	NA	40^{+4}	Live birth	No	No	3235	None	Neonatal oral swab	Negative	No
Chen et al. (21)	20 Jan - 10 Feb 2020<br><br>China	1 case	30	Pre-eclampsia	Caesarean	Fetal tachycardi a	39^{+1}	Live birth	No	No	3800	None	Neonatal oral swab	Negative	No
Chen et al. (21)	20 Jan - 10 Feb 2020<br><br>China	1 case	25	None	Vaginal	NA	38^{+6}	Live birth	No	No	3670	None	Neonatal oral swab	Negative	No
Chen et al. (21)	20 Jan - 10 Feb 2020<br><br>China	1 case	31	None	Vaginal	NA	39^{+6}	Live birth	No	No	3700	None	Neonatal oral swab	Negative	No
Chen et al. (21)	20 Jan - 10 Feb 2020<br><br>China	1 case	29	Gestational diabetes	Caesarean	Diabetes gestational	39^{+0}	Live birth	No	No	4050	None	Neonatal oral swab	Negative	No

Chen et al. (37)	Published on 16 Mar 2020<br><br>China	1 case	28	None	Caesarean	COVID-19 pneumonia (performed in the acute phase)	37^{+2}	Live birth	No	No	3200	None	Neonatal oral swab (72h after birth)	Negative	No
Chen et al. (37)	Published on 16 Mar 2020<br><br>China	1 case	34	Cholecystitis	Caesarean	COVID-19 pneumonia (performed in the acute phase)	39^{+0}	Live birth	No	No	3050	NICU admission: Edema, rash (disappeared spontaneously without treatment).	Not tested (without parent´s consent)	Not tested	No
Chen et al. (37)	Published on 16 Mar 2020<br><br>China	1 case	23	Anemia, placenta previa	Caesarean	COVID-19 pneumonia (performed in the acute phase), placenta previa	37^{+3}	Live birth	NI<br><br>(severe dyspnea after delivery which required respiratory support)	No	3800	NICU admission: Mild dyspnea and transient tachypnea (non-invasive mechanical ventilation for 3 days), edema, rash (disappeared spontaneously without treatment).	Neonatal oral swab (72h after birth)	Negative	No
Chen et al. (37)	Published on 16 Mar 2020<br><br>China	1 case	31	Reduced fetal movement	Vaginal (emergency labor process)	NA	38^{+4}	Live birth	No	No	3550	None	Neonatal oral swab (72h after birth)	Negative	No

Collin et al. (156)	19 Mar - 20 Apr 2020<br><br>Sweden	13 cases	20-35	Gestational diabetes and obesity (n=NI)	Caesarean (n=5)<br><br>Vaginal (n=2)<br><br>Not informed delivered/ undelivered (n=6)	Obstetric indication (n=2), COVID-19 (n=2), NI (n=1)	13^{+0}-40^{+0}	NI	Yes (n=13)<br><br>(invasive mechanical ventilation (n=7))	No	NI	NI	NI	NI	NI
Cooke et al. (23)	Published on 15 May 2020<br><br>UK	1 case	39	Type 2 diabetes mellitus and obesity (BMI=42 Kg/m^2)	Caesarean	Clinical condition (to facilitate invasive ventilation)	28^{+5}	Live birth	Yes (Invasive Ventilation)	No	1530	Newborn intubated shortly after birth, diagnosed with a spontaneous bowel perforation on day 6 and transferred to a tertiary unit	RT-PCR in neonatal sample (not specified)	Negative	No
Cooke et al. (23)	Published on 15 May 2020<br><br>UK	1 case	28	Gestational diabetes	Caesarean	Clinical condition (maternal respiratory deterioration)	29^{+0}	Live birth	NI (Invasive ventilation, intubated for caesarean 42h after admission and extubated 7h post-operatively)	No	1400	No	RT-PCR in neonatal sample (not specified)	Negative	No
Cosma et al. (169)	22 Fev-21 May 2020<br><br>Italy	23 cases	NI	NI	Case group: spontaneous abortion (n=11)<br><br>Control group:	NA	Case group: in first trimester<br><br>Control group: at 12 gestational weeks	Spontaneous abortions (n=11)<br><br>Pregnant (n=12)	No	No	NA	NA	NA	NA	NA

					ongoing pregnancy (n=12)										
Deng et al. (38)	28 Jan-28 Feb 2020<br><br>China	37 cases	Pregnant patients without liver injury (n=26): 30.46±4.09<br><br>Pregnant patients with liver injury (n=11): 31.18±5.31	Pregnant patients without liver injury (n=26): Cardiovascular disease (n=1)<br><br>Pregnant patients with liver injury (n=11): Hypertension (n=1); diabetes (n=1)	Caesarean (n=4)<br><br>Vaginal (n=2)<br><br>Pregnant (n=31)	Mother request CS (n=4)	2nd trimester (n=5)<br><br>3rd trimester (n=4)	Live births (n=6)	No	No	NI	None	RT-PCR in breastmilk (n=6), neonatal throat swab (n=4), neonatal anal swab (n=1)	Negative (n=6)	No (n=6)
Dong et al. (77)<br><br>Wu et al. (148)	2 Feb – 18 Mar 2020 China	1 case	29	None	Caesarean	COVID-19 potential mother-to-child transmission	37^{+6}	Live birth	No	No	3120	Suspected pneumonia	Neonatal throat and anal swabs (1st and 3rd days after birth); vaginal secretions sample; neonatal blood breastmilk	Negative RT-PCR (breast milk; nasopharyngeal swabs in 5 samples collected between 2h after birth to day 16 after birth)<br><br>Elevated levels of SARS-CoV-2 IgM e IgG	No

														(day of birth and day 15 after birth)	
Dória et al. (24)	25 Mar-15 Apr 2020<br><br>Portugal	1 case	22	Fetal growth restriction	Pregnant	NA	GA at diagnosis 35^{+0}	NA	No	No	NA	NA	NA	NA	NA
Dória et al. (24)	25 Mar-15 Apr 2020<br><br>Portugal	1 case	41	Ulcerative colitis, Psoriasis (chronic corticotherapy), PROM	Pregnant	NA	GA at diagnosis 30^{+0}	NA	No	No	NA	NA	NA	NA	NA
Dória et al. (24)	25 Mar-15 Apr 2020<br><br>Portugal	1 case	36	Severe scoliosis, Behçet syndrome, fetal growth restriction	Caesarean	NI	GA at diagnosis 37	Live birth	No	No	2350	No	RT-PCR in neonatal sample (not specified)	Negative	No
Dória et al. (24)	25 Mar-15 Apr 2020<br><br>Portugal	1 case	38	Gestational diabetes, fetal growth restriction	Caesarean	NI	GA at diagnosis 39	Live birth	No	No	2480	No	RT-PCR in neonatal sample (not specified)	Negative	No

Dória et al. (24)	25 Mar-15 Apr 2020 Portugal	1 case	27	None	Caesarean	NI	GA at diagnosis 41	Live birth	No	No	3380	No	RT-PCR in neonatal sample (not specified)	Negative	No
Dória et al. (24)	25 Mar-15 Apr 2020 Portugal	1 case	32	Gestational hypertension	Vaginal	NA	GA at diagnosis 37	Live birth	No	No	2600	No	RT-PCR in neonatal sample (not specified)	Negative	No
Dória et al. (24)	25 Mar-15 Apr 2020 Portugal	1 case	33	Severe myopia, Fetal growth restriction	Caesarean	NI	GA at diagnosis 40	Live birth	No	No	2670	No	RT-PCR in neonatal sample (not specified)	Negative	No
Dória et al. (24)	25 Mar-15 Apr 2020 Portugal	1 case	34	Fetal growth restriction	Vaginal	NA	GA at diagnosis 40	Live birth	No	No	2795	No	RT-PCR in neonatal sample (not specified)	Negative	No
Dória et al. (24)	25 Mar-15 Apr 2020 Portugal	1 case	35	Asthma, Raynaud syndrome, fetal growth restriction	Vaginal	NA	GA at diagnosis 37	Live birth	No	No	2410	No	RT-PCR in neonatal sample (not specified)	Negative	No
Dória et al. (24)	25 Mar-15 Apr 2020 Portugal	1 case	27	None	Vaginal	NA	GA at diagnosis 39	Live birth	No	No	3220	No	RT-PCR in neonatal sample (not specified)	Negative	No

Dória et al. (24)	25 Mar- 15 Apr 2020<br><br>Portugal	1 case	29	Chronic hypertension, fetal growth restriction	Caesarean	NI	GA at diagnosis 38	Live birth	No	No	2580	No	RT-PCR in neonatal sample (not specified)	Negative	No
Dória et al. (24)	25 Mar- 15 Apr 2020<br><br>Portugal	1 case	29	Dichorionic Diamniotic Pregnancy, Fetal growth discordance	Caesarean	NI	GA at diagnosis 37	Live birth (n=2, twin pregnancy)	No	No	Twin 1: 2370; Twin 2: 2745	No	RT-PCR in neonatal sample (not specified)	Negative	No
Du et al. (93)	Published on 19 May 2020<br><br>China	1 case	30	None	Caesarean	Fetal distress (emergenc y caesarean section)	37^{+1}	Live birth	No	No	2730	None	RT-PCR in neonatal sample (not specified)	Negative	No
Fan et al. (25)	17 Jan – 19 Feb 2020<br><br>China	1 case	29	Vaginal bleeding, COVID-19 pneumonia	Caesarean	Persistent fever	36^{+5}	Live birth	No	No	2890	Mild neonatal pneumonia and lymphopenia	Placenta, cord blood, vaginal swab, amniotic fluid, breast milk. Newborn´ s nasophary ngeal swabs	Negative	No
Fan et al. (25)	17 Jan – 19 Feb 2020<br><br>China	1 case	34	COVID-19 pneumonia	Caesarean	NI	37^{+0}	Live birth	No	No	3400	Low-grade fever and abdominal distension with lymphopenia on day 3.	Placenta, cord blood, vaginal swab, amniotic fluid,	Negative	No

												Lung infection.	breast milk. Newborn´s nasopharyngeal swabs at birth		
Ferrazzi et al. (136)<br><br>Ferrazzi et al. (157)	1-20 Mar 2020<br><br>Italy	42 cases	Mean: 32.9 (range: 21-44)	Gestational diabetes (n=6); COVID-19 pneumonia (n=19)	Caesarean (n=18)<br><br>Vaginal (n=24)	Clinical condition (worsening dyspnea or other COVID-19 related symptoms) (n=10); obstetric reasons unrelated to COVID-19 infection (n=8)	>37 (n=30); >34-37 (n=7); ≤34 (n=4)<br><br>Preterm (n=11) Missing (n=1)	Live birth (n=42)	Yes (n=4)<br><br>Required oxygen support (n=7)	No (n=42)	Elective CS for COVID (n=10) Mean: 2730 (840–4040);<br><br>Elective CS for obstetric reasons (n=8) Mean: 3100 (2770–3430);<br><br>vaginal delivery (n=24) Mean: 3226 (2450–3740)	NICU admissions for preterm birth/respiratory distress (n=3).<br><br>One term neonate developed gastrointestinal symptoms within a few hours after a vaginal birth; after 3 days, he developed respiratory symptoms and was transferred to the NICU, where he recovered after 1 day of mechanical ventilation (The first test for SARS-CoV-2 was equivocal a few hours after delivery, but positive 3 days later).	RT-PCR in neonatal sample (not specified)	Positive (n=3):<br><br>[2 in women with diagnosis after delivery and that breastfed without mask, newborns with positive test for COVID-19 infection at day 1 and 3); 1 after a vaginal delivery (1st test a few hours after delivery was equivocal but positive 3 days later)	No (n=42)

Fox et al. (26)	22 Mar-30 Apr 2020<br><br>USA	33 cases	Mean: 31.0±6.2	Obesity (n=1); diabetes type 1 (n=1); hypothyroidism (n=1)	Delivered, type of birth not informed (n=17)<br><br>Abortion (n=1)<br><br>Pregnant (n=15)	NI	Abortion GA 14 weeks (n=1)<br><br>GA not informed (n=17)	Live birth (n=17)<br><br>Abortion (n=1)	No (n=33, without mechanical ventilation)<br><br>Oxygen therapy (n=1)	No (n=33)	NI	NI	NI	NI	NI
Giannini et al. (39)	Published on May 2020<br><br>Italy	17 cases	NI	NI	NI	NI	NI	NI	No	No	NI	NI	NI	NI	NI
Giannini et al. (39)	Published on May 2020<br><br>Italy	4 cases	29-35	COVID-19 pneumonia	Caesarean section (n=2, at 33 and 38 weeks of GA)	NI	21, 25, 33 and 38 weeks of gestation	NI	Yes (n=4)<br><br>(intubation and mechanical ventilation=2; non-invasive ventilation via face masks=2)	No	NI	NI	NI	NI	NI
Gidlöf et al. (94)	Published on 6 April 2020<br><br>Sweden	1 case	34	Preeclampsia, gestational diabetes, dichorionic twin pregnancy	Caesarean	NI	36^{+2}	Live birth (n=2)	No	No	Twin1: 2680; Twin2: 2160	Twin1-gastrointestinal reflux	Neonatalnasopharyngeal swab (34 hours and 4 days after birth); breast milk and maternal vaginal secretion (5 days after birth)	Negative	No

Govindel al. (40)	7 Mar - 22 Apr 2020 UK	1 case	36	Gestational diabetes; COVID-19 pneumonia	Caesarean	Deterioration of maternal respiratory function - respiratory distress (emergency CS)	38	Live birth	NI	No	4165	Viral pneumonia day 6	Neonatal nasopharyngeal swab samples	Positive	No
Govindel al. (40)	7 Mar - 22 Apr 2020 UK	1 case	29	COVID-19 pneumonia	Caesarean	Deterioration of maternal respiratory function - respiratory distress (emergency CS)	27	Live birth	NI	No	1200	Ventilated for 10 days	Neonatal nasopharyngeal swab samples	Negative	No
Govindel al. (40)	7 Mar - 22 Apr 2020 UK	1 case	31	None	Caesarean	Pathological CTG not in labor (emergency CS)	35	Live birth	NI	No	2700	Poor feeding	Neonatal nasopharyngeal swab samples	Negative	No
Govindel al. (40)	7 Mar - 22 Apr 2020 UK	1 case	31	Asthma	Caesarean	Breech (elective CS)	39	Live birth	NI	No	3370	Talipes	Neonatal nasopharyngeal swab samples	Negative	No
Govindel al. (40)	7 Mar - 22 Apr 2020 UK	1 case	22	None	Vaginal	NA	38	Live birth	NI	No	4300	None	Neonatal nasopharyngeal swab samples	Negative	No

Govind el al. (40)	7 Mar - 22 Apr 2020<br><br>UK	1 case	39	Insulin dependent Diabetes Hypertension	Caesarean	3 previous caesarean section (elective CS)	37	Live birth	NI	No	2500	None	Neonatal nasophary ngeal swab samples	Negative	No
Govind el al. (40)	7 Mar - 22 Apr 2020<br><br>UK	1 case	18	None	Caesarean	Previous caesarean section and maternal request (elective CS)	39	Live birth	NI	No	3060	None	Neonatal nasophary ngeal swab samples	Negative	No
Govind el al. (40)	7 Mar - 22 Apr 2020<br><br>UK	1 case	38	None	Caesarean	3 previous caesarean section (elective CS)	39	Live birth	NI	No	3540	None	Neonatal nasophary ngeal swab samples	Negative	No
Govind el al. (40)	7 Mar - 22 Apr 2020<br><br>UK	1 case	34	None	Caesarean	Maternal request (elective CS)	39	Live birth	NI	No	3560	None	Neonatal nasophary ngeal swab samples	Negative	No
Gulersen et al. (158)<br><br>Blitz. et al. (35)	13 Mar-24 Apr 2020<br><br>USA	67 cases	Range: 29-35	Multiple fetal structural malformations detected prior to SARS-CoV-2 infection (n=2); fetal growth restriction (n=3); PPROM (n=7); preeclampsia (n=3); choriomnionitis (n=1); placenta previa (n=1);	Delivered, type of birth not informed (n=49)<br><br>Pregnant (n=18)	NI	Early preterm at diagnosis ($23-33^{+6}$) (n=36/65)<br><br>Late preterm at diagnosis ($34-36^{+6}$) (n=29/65)<br><br>Stillbirth (36 and 35^{+4}	Live birth (n=47)<br><br>Stillbirth (n=2)	Yes (n=5)	No	NI	NI	NI	NI	NI

				placenta accreta (n=1)			weeks) (n=2/67)								
Griffin et al. (137)	31 Mar-5 May 2020<br><br>USA	27 cases	NI	HELLP syndrome or pregnancy induced hypertension (n=3)	Vaginal (n=17)<br><br>Caesarean (n=10)	Repeat CS (n=5); primary CS (n=3); emergent CS (n=2)	Mean: 38.9±1.5	Live birth (n=27)	NI	No	Mean: 3317±501	NICU admission (n=2)<br><br>No infants had clinical evidence of symptomatic COVID-19 infection.<br><br>Respiratory distress (n=1); hypoxic ischemic encephalopathy requiring whole body cooling (n=1); Failed hearing screen (unilateral) (n=1); Hypoglycemia requiring one glucose gel (n=1)	Neonatal nasopharyngeal swab (24h after birth) (n=14)	Negative (n=14)	No
Hansen et al. (120)	Published on 14 May 2020<br><br>USA	1 case	37	Diabetes mellitus; obesity; vaginal bleeding	Ectopic pregnancy	NA	NI	Ectopic pregnancy	No	No	NA	NA	NA	NA	NA

Hantoushzadeh et al. (41)	Feb - Mar 2020<br><br>Iran	1 case	25-29	ARDS	Vaginal	NA	30^{+3}	Stillbirth	Yes (mechanical ventilation)	Yes	1700	NA	NA	NA	NA
Hantoushzadeh et al. (41)	Feb - Mar 2020<br><br>Iran	1 case	25-29	Obesity; ARDS; fetal distress	Caesarean	Fetal distress	38^{+3}	Live birth	Yes (mechanical ventilation)	Yes	2800	None	Neonatal nasopharyngeal swab samples	Negative	No
Hantoushzadeh et al. (41)	Feb - Mar 2020<br><br>Iran	1 case	40-44	Subclinical hypothyroid; ARDS	Caesarean	Emergency caesarean delivery; decreased fetal movement and acutely decompensated	30^{+5}	Live birth	Yes (mechanical ventilation)	Yes	2100	Neonatal pneumonia Neonatal lymphopenia	Neonatal nasopharyngeal swab	Negative (day 1)<br><br>Positive (day 7)	No
Hantoushzadeh et al. (41)	Feb - Mar 2020<br><br>Iran	1 case	30-34	Dichorionic diamniotic twin gestation; ARDS; renal failure	Maternal death before delivery	NA	24^{+0}	Stillbirth	Yes (mechanical ventilation)	Yes	NA	NA	NA	NA	NA
Hantoushzadeh et al. (41)	Feb - Mar 2020<br><br>Iran	1 case	30-34	Gestational diabetes	Caesarean	Risk of cardiopulmonary collapse	36^{+0}	Live birth	Yes (mechanical ventilation)	Yes	3200	None	Neonatal nasopharyngeal swab	Negative	No

Hantous hzadeh et al. (41)	Feb - Mar 2020 Iran	1 case	35-39	ARDS	Maternal death before delivery	NA	24^{+0}	Stillbirth (n=2; twin pregnancy)	Yes (mechanical ventilation)	Yes	NA	NA	NA	NA	NA
Hantous hzadeh et al. (41)	Feb - Mar 2020 Iran	1 case	45-49	Underweight; Dichorionic diamniotic twin gestation; ARDS; intermittent hypoxemia; fetal distress	Caesarean	Fetal distress	28^{+0}	Live birth (n=2; twin pregnancy)	Yes (mechanical ventilation)	Yes	T1 1180 T2 1340	None	Neonatal nasophary ngeal swab (n=2)	Negative (n=2)	Yes (n=2)
Hantous hzadeh et al. (41)	Feb - Mar 2020 Iran	1 case	35-39	Obesity	Caesarean	Fetal tachycardi a; breech presentati on	33^{+5}	Live birth	Yes (mechanical ventilation)	No	1800	None	Neonatal nasophary ngeal swab	Negative	No
Hantous hzadeh et al. (41)	Feb - Mar 2020 Iran	1 case	35-39	Gestational diabetes; obesity; PPROM; coagulopathy	Caesarean	Breech presentati on	36^{+0}	Stillbirth	Yes (mechanical ventilation)	No	3000	NA	NA	NA	NA
Hijona et al. (60) Hijona et al. (61)	Published on 5 Jun 2020 Spain	1 case	31	Suspected chromosomal abnormality in fetus	Pregnant	NA	GA at diagnosis 16	NA	No	No	NA	NA	Vaginal fluid, amniotic fluid	Negative	NA
Hijona et al. (60) Hijona et al. (61)	Published on 5 Jun 2020 Spain	1 case	39	Suspected chromosomal abnormality in fetus	Pregnant	NA	GA at diagnosis 16	NA	No	No	NA	NA	Vaginal fluid, amniotic fluid	Negative	NA

Hijona et al. (60) Hijona et al. (61)	Published on 5 Jun 2020 Spain	1 case	27	Suspected chromosomal abnormality in fetus	Pregnant	NA	GA at diagnosis 21	NA	No	No	NA	NA	Vaginal fluid, amniotic fluid	Negative	NA
Hijona et al. (60) Hijona et al. (61)	Published on 5 Jun 2020 Spain	1 case	40	Suspected infection by Cytomegalovirus	Pregnant	NA	GA at diagnosis 24	NA	No	No	NA	NA	Vaginal fluid, amniotic fluid	Negative	NA
Hirshberg et al. (62)	Published on 1 May2020 USA	1 case	29	Chronic kidney disease; hypertension; COVID-19 pneumonia; ARDS	Caesarean	Maternal indication (concern for further decompensation)	31^{+4}	Live birth	Yes (intubated, mechanical ventilation)	No	1500	NI	RT-PCR in neonatal sample (not specified) (24h after birth)	Negative	No
Hirshberg et al. (62)	Published on 1 May 2020 USA	1 case	33	Mild intermittent asthma; COVID-19 pneumonia; ARDS; septic shock	Pregnant	NA	GA at admission 26^{+0}	NA	Yes (intubated, mechanical ventilation)	No	NA	NA	NA	NA	NA
Hirshberg et al. (62)	Published on 1 May 2020 USA	1 case	39	Obesity; hypertension; insulin-dependent diabetes; obstructive sleep apnea: COVID-19 pneumonia; ARDS	Caesarean	Maternal indication (critical illness)	30^{+2}	Live birth	Yes (intubated, mechanical ventilation)	No	2110	NI	RT-PCR in neonatal sample (not specified) (24h after birth)	Negative	No
Hirshberg et al. (62)	Published on 1 May 2020 USA	1 case	27	Hypertension; obesity; COVID-19 pneumonia	Caesarean	Maternal indication (declining respiratory status)	31^{+3}	Live birth	Yes (intubated, mechanical ventilation)	No	1845	NI	RT-PCR in neonatal sample (not specified)	Negative	No

													(24h after birth)		
Hirshberg et al. (62)	Published on 1 May 2020<br><br>USA	1 case	35	Obesity; COVID-19 pneumonia	Pregnant	NA	GA at admission 25^{+2}	NA	Yes<br><br>(intubated, mechanical ventilation)	No	NA	NA	NA	NA	NA
Hong et al. (95)	Published May 5<br><br>USA	1 case	36	Hypothyroidism; Morbid obesity; Hyperlipidemia	Pregnant	NA	GA at admission 23^{+0}	NA	Yes (mechanical ventilation)	No	NA	NA	NA	NA	NA
Hosier et al. (121)	Mar 2020<br><br>USA	1 case	35	Psoriasis, severe hypertension, severe thrombocytopenia, placental abruption, intravascular coagulopathy; severe preeclampsia, vaginal bleeding	Termination of the pregnancy performed via dilation and evacuation	To reduce the risk of serious maternal morbidity or death	22	Medical termination of the pregnancy	Yes<br><br>(intubated)	No	NA	NA	Placenta, cord blood<br><br>Fetal samples: heart and lungs	Positive (Placenta, cord blood)<br><br>Negative (Fetal samples)	NA
Hu et al. (42)<br><br>Liu et al. (64)	20 Jan - 20 Feb<br><br>China	1 case	34	COVID-19 pneumonia	Caesarean	To reduce the risk of vertical transmission	40	Live birth	No	No	3250	None	Amniotic fluid samples; neonatal throat swabs at 24-36h of life as well as blood,	Negative<br><br>Positive (Throat swab at 36 hours of life)	No

													urine, and feces		
Hu et al. (42)<br>Liu et al. (64)	20 Jan - 20 Feb<br>China	1 case	31	COVID-19 pneumonia	Caesarean	To reduce the risk of vertical transmission	41^{+2}	Live birth	No	No	3470	None	Amniotic fluid samples; neonatal throat swabs at 24-36h of life as well as blood, urine, and feces	Negative	No
Hu et al. (42)<br>Liu et al. (64)	20 Jan - 20 Feb<br>China	1 case	34	Liver dysfunction; COVID-19 pneumonia	Caesarean	To reduce the risk of vertical transmission	38^{+4}	Live birth	No	No	3250	None	Amniotic fluid samples; neonatal throat swabs at 24-36h of life as well as blood, urine, and feces	Negative	No
Hu et al. (42)<br>Liu et al. (64)	20 Jan - 20 Feb<br>China	1 case	30	COVID-19 pneumonia	Vaginal	NA	39^{+5}	Live birth	No	No	3670	None	Amniotic fluid samples; neonatal throat swabs at 24-36h of life as well as blood, urine, and feces	Negative	No

Hu et al. (42)<br><br>Liu et al. (64)	20 Jan - 20 Feb<br><br>China	1 case	33	PROM; COVID-19 pneumonia	Caesarean	To reduce the risk of vertical transmission	38^{+2}	Live birth	No	No	3180	None	Amniotic fluid samples; neonatal throat swabs at 24-36h of life as well as blood, urine, and feces	Negative	No
Hu et al. (42)<br><br>Liu et al. (64)	20 Jan - 20 Feb<br><br>China	1 case	34	COVID-19 pneumonia	Caesarean	To reduce the risk of vertical transmission	38^{+2}	Live birth	No	No	3200	None	Amniotic fluid samples; neonatal throat swabs at 24-36h of life as well as blood, urine, and feces	Negative	No
Hu et al. (42)<br><br>Liu et al. (64)	20 Jan - 20 Feb<br><br>China	1 case	33	COVID-19 pneumonia	Caesarean	To reduce the risk of vertical transmission	37^{+2}	Live birth	No	No	3300	None	Amniotic fluid samples; neonatal throat swabs at 24-36h of life as well as blood, urine, and feces	Negative	No
Huang et al. (43)	20 Jan - 20 Feb<br><br>China	1 case	29	Mild anemia	Pregnant	NA	GA at admission 30^{+0}	NA	No	No	NA	NA	NA	NA	NA

Huang et al. (43)	20 Jan - 20 Feb China	1 case	27	None	Pregnant	NA	GA at admission 34^{+0}	NA	No	No	NA	NA	NA	NA	NA
Huang et al. (43)	20 Jan - 20 Feb China	1 case	28	None	Caesarean	NI	39^{+3}	Live birth	No	No	4200	No	Neonatal throat swab	Negative	No
Huang et al. (43)	20 Jan - 20 Feb China	1 case	33	Uterine scarring	Caesarean	NI	38^{+4}	Live birth	No	No	2367	No	Neonatal throat swab	Negative	No
Huang et al. (43)	20 Jan - 20 Feb China	1 case	29	Mild anemia; Fetal distress	Caesarean	Emergent CS; Fetal distress	37^{+5}	Live birth	No	No	2585	No	Neonatal throat swab	Negative	No
Huang et al. (43)	20 Jan - 20 Feb China	1 case	29	Heart and respiratory failure, mild anemia, eclampsia, PROM; Fetal distress	Vaginal	Fetal distress	31^{+2}	Live birth (n=2; twin pregnancy)	Yes (no information on mechanical ventilation)	No	T1:1520 T2:1720	NICU admission (n=2) T1: severe neonatal asphyxia; T2: suspected viral pneumonia; severe neonatal asphyxia	Neonatal throat swab	Negative	Yes (T1) No (T2)

Huang et al. (43)	20 Jan - 20 Feb<br><br>China	1 case	32	Uterine scarring; septic shock, SCIM, ARDS, MODS; Fetal distress	Caesarean	Emergent CS; Fetal distress	35^{+2}	Live birth	Yes (mechanical ventilation; ECMO)	No	2700	Severe neonatal asphyxia (Apgar 1,1)	Not tested	NA	Yes
Huang et al. (43)	20 Jan - 20 Feb<br><br>China	1 case	32	Moderate anemia; fetal distress	Caesarean	Emergent CS; Fetal distress	28^{+1}	Live birth	Yes (no information on mechanical ventilation)	No	1530	NICU admission; severe neonatal asphyxia	Neonatal throat swab	Negative	No
Iqbal et al. (78)	Published on 1 April 2020<br><br>USA	1 case	34	None	Vaginal	NA	39^{+3}	Live birth	No	No	NI	None	Amniotic fluid, vaginal side walls, and rectum. Neonatal swabs: nasopharynx, oropharynx, oral mucosa, skin surface and rectum right after delivery	Negative	No
Inchingolo et al. (122)	10 Mar 2020<br><br>Italy	1 case	NI	COVID-19 pneumonia	Pregnant	NA	GA at admission 23^{+0}	NA	No	No	NA	NA	NA	NA	NA

Indraccolo et al. (123)	9 Mar 2020<br><br>Italy	1 case	NI	Epigastralgia	Pregnant	NA	GA at admission 32^{+0}	NA	No	No	NA	NA	NA	NA	NA
Jain et al. (44)	Published 27 May<br><br>India	1 case	NI	Fetal distress	Caesarean	Emergency CS; Fetal distress	Term; 3rd trimester	Live birth	NI	No	2865	None	RT-PCR in neonatal sample (not specified)	Negative	No
Jain et al. (44)	Published 27 May<br><br>India	1 case	NI	Fetal distress	Caesarean	Fetal distress; meconium stained liquor	Term; 3rd trimester	Live birth	NI	NI	NI	Resuscitation and mechanical ventilation; hypoxic ischemic encephalopathy	Neonatal nasopharyngeal swabs (day 3, 5, 8 after birth)	Negative	No
Joudi et al. (124)	Published on May 2020<br><br>USA	1 case	26	Preeclampsia	Vaginal	NA	37 weeks	Live birth	No	No	3042	NICU admission; penile torsion	NI	Negative	No
Juusela et al. (63)	Mar-April 2020<br><br>USA	1 case	45	Gestational diabetes, obesity, preeclampsia; acute heart failure; cardiopulmonary resuscitation	Caesarean	Clinical condition<br><br>(to aid maternal resuscitation)	39^{+3}	Live birth	Yes (mechanical ventilation)	No	NI	NI	NI	NI	No
Juusela et al. (63)	Mar-April 2020<br><br>USA	1 case	26	Obesity, polycystic ovary syndrome, reduced fetal movement;	Caesarean	COVID-19 pneumonia	34^{+3}	Live birth	No	No	NI	NI	NI	NI	No

Kalafat et al. (96)	19 -29 Mar 2020<br><br>Turkey	1 case	32	Thalassemia trait; COVID-19 pneumonia	Caesarean	COVID-19; reduced tidal volume from the increased abdominal pressure	36^{+1}	Live birth	Yes (intubated)	No	2790	None	Placenta, cord blood, neonatal throat and nasal swabs, breast milk	Negative	No
Kang et al. (125)	Feb 2020<br><br>China	1 case	30	Fetal distress	Caesarean	Fetal intrauterine distress, fetal tachycardia	35^{+4}	Live birth	NI	No	NI	None	Neonatal pharyngeal swab, amniotic fluid, cord blood and placenta	Negative	No
Kayem et al. (45)	1 Mar – 14 Apr 2020<br><br>France	617 cases	≤35 years (n= 423)<br><br>>35 years (n=194)	Obesity (n=139); asthma (n=37); chronic respiratory disease (n=6); diabetes type1 or 2 (n=14); chronic hypertension (n=18); gestational diabetes (n=71); gestational hypertension or preeclampsia (n=21)	Caesarean (n=87)<br><br>Vaginal (n=94)<br><br>Pregnant (n=436)	COVID-19 symptoms (n=45)	Preterm births (22-31 weeks) (n=21)<br><br>Preterm births (32-36 weeks) (n=29)<br><br>Term births (n=131)	Spontaneous abortion (14-21 weeks) (n=5)<br><br>Stillbirth (n=7)<br><br>Live birth (n=178; 7 twin pregnancies; triple pregnancy)	Yes (n=35) (non-invasive mechanical (n=10); invasive mechanical ventilation (n=29); ECMO (n=6); oxygen therapy (n=83))	Yes (n=1)	NI	Admission to NICU (n=37)	RT-PCR in neonatal sample	Negative (n=176)<br><br>Positive (n=2)	No (n=177)<br><br>Yes (n=1)
Kelly et al. (126)	Published on 28 Apr 2020<br><br>USA	1 case	NI	Obesity	Caesarean	Clinical condition (Persistent maternal tachycardia and high fever)	GA at hospital admission 33^{+0}	Live birth	Yes (intubated)	No	NI	NI	RT-PCR in neonatal sample (not specified) (5 days after birth)	Negative	No

Khan et al.<br>(46)	25 Jan - 15 Feb 2020<br><br>China	17 cases	29.29 (range: 24-34)	PROM (n=NI)	Caesarean (n=17)	Emergency caesarean section due to PROM (n=NI, “some of the women”)	Mean: 38.1 (Range: 35^{+5}-41)<br><br>Preterm delivery (n=3)	Live births (n=17)	NI	No (n=17)	3104.375 (range: 2300-3750)	Neonatal pneumonia (n=5)	Neonatal throat swab samples	Negative (n=15)<br><br>Positive samples tested within 24 hours after the delivery (n=2)	No (n=17)
Khan et al.<br>(47)	28 Jan - 1 Mar 2020<br><br>China	1 case	28	COVID-19 pneumonia	Vaginal	NA	34^{+6}	Live birth	NI	No	2890	None	Neonatal throat swab (12 hours after delivery)	Negative	No
Khan et al.<br>(47)	28 Jan - 1 Mar 2020<br><br>China	1 case	33	COVID-19 pneumonia	Vaginal	NA	39^{+1}	Live birth	NI	No	3500	None	Neonatal throat swab (12 hours after delivery)	Negative	No
Khan et al.<br>(47)	28 Jan - 1 Mar 2020<br><br>China	1 case	27	COVID-19 pneumonia	Vaginal	NA	38^{+2}	Live birth	NI	No	3730	None	Neonatal throat swab (12 hours after delivery)	Negative	No
Khoury et al<br>(138)	13 Mar – 12 Apr 2020<br><br>USA	241 cases	Median: 32 (range: 18-47)	Obesity (n=98); PROM (n=41); COVID-19 pneumonia (n=43)	Cesarean (n=100)<br><br>Vaginal (n=141)	Worsening maternal respiratory status (n=10); severe/critical COVID-19 (n=44); Nonreassuring fetal heart rate	Mean GA: 39.0 (range: 24.7-41.6)<br><br>Preterm (n=34)	Live birth (n=245; 6 twin pregnancies)<br><br>Stillbirths (n=2)	Yes (n=17; 9 with maternal intubation)	No (n=241)	Median: 3135 (range 640-4700)	Resuscitation at delivery (n=70); NICU admission (n=61); respiratory distress syndrome (n=14); complications of prematurity	Nasopharyngeal swab at 24h of life and 96h of life or discharge (n=236)	Negative (n=230)<br><br>Positive (n=6)	No (n=241)

						tracing (n=23); failed induction (n=11); active phase arrest (=5); arrest of descent (=5); malpresentation (n=5); repeat CS (n= 31); other indication (n=10)						or low birthweight (n=21); sepsis (n=1); congenital anomaly (n=8); others (n=14)			
Kirtsman et al. (97)<br><br>Koumoutsea et al. (55)	Published May 14<br><br>Canada	1 case	40	Familial neutropenia, gestational diabetes and a history of frequent bacterial infections, including 3 episodes (sinusitis, skin infection and bronchitis) during the current pregnancy; acute coagulopathy	Caesarean	Semi-urgent CS, owing to worsening coagulopathy	35^{+5}	Live birth	NI	No	2930	NICU admission<br><br>(The neonate was noted to be neutropenic and had mild hypothermia, feeding difficulties and intermittent hypoglycemic episodes)	Placental swabs (both maternal and fetal sides); umbilical cord tissue; neonatal nasopharyngeal swabs (at birth, day 2 and day 7); neonatal plasma (day 1, 2, 4 and 7); neonatal stool sample at	Negative (umbilical cord)<br><br>Positive (placental swabs; for the 3 nasopharyngeal swabs samples; neonatal stool at day 7; breast milk at day 2)	No

													day 7; breast milk (day 2 and day 7)		
Kleinwechter et al. (127)	Mar 2020<br><br>Germany	1 case	28	Gestational diabetes	Pregnant	NA	GA at admission 25^{+6}	NA	NI	No	NA	NA	NA	NA	NA
Knight et al. (139)	March 1 to April 14, 2020<br><br>UK	427 cases	<20 (n=4);<br><br>20-34 (n=248);<br><br>≥ 35 (n=175)	Overweight (n=141); obese (n=140); gestational diabetes (n=50); asthma (n=31); diabetes (n=13); hypertension (n=12); cardiac disease (n=6); COVID-19 pneumonia (n=104)	Miscarriage (n=4)<br><br>Caesarean (n=156)<br><br>Vaginal (n=106)<br><br>Pregnant (n=161)	CS by maternal indication due to COVID-19 (n=42)<br><br>CS by other indications (n=114): fetal compromise (n=37); failure to progress in labor or induction (n=30); other obstetric reasons (n=25); previous CS (n=16); maternal request (n=6)	Miscarriage (n=4/266): 10-19 weeks<br><br>GA at birth (n=262/266): 22-27 weeks (n=6); 28-31 weeks (n=17); 32-36 weeks (n=43); ≥37 weeks (n=196)<br><br>Preterm delivery (n=66/262)	Miscarriage (n=4)<br><br>Stillbirth (n=3)<br><br>Live birth (n=265, 6 twin pregnancy)	Yes (n=41/427) (Respiratory support, not specified (n=41); ECMO (n=4))	Yes (n=5/427)<br><br>Still in hospital (n=25/427)	NI	NICU admission (n=67/265)<br><br>Neonatal encephalopathy grade1 (n=1/265)	Neonatal RT-PCR of blood or a nasopharyngeal swab or aspirate	Negative (n=253/265)<br><br>Positive (n=12/265): <12 hours (n=6), ≥12 hours (n=6)	Yes (n=2)

Koumoutsea et al (55)	Published April 17<br><br>Canada	1 case	23	Asthma; obesity; acute coagulopathy	Caesarean	Emergency CS due to non-reassuring fetal heart rate coupled with progressive coagulopathy and transaminitis	35^{+5}	Live birth	NI	No	2540	NI	NI	NI	No
Kuhrt et al. (98)	28 Mar 2020<br><br>UK	1 case	30	Thyroidectomy after a papillary cell carcinoma; antepartum hemorrhage; placental abruption	Caesarean	Emergency CS owing to antepartum hemorrhage	32^{+6}	Live birth (n=2, twin pregnancy)	No	No	T1: 2190 T2: 2160	NICU admission (n=2); required positive pressure respiratory resuscitation	RT-PCR in neonatal sample (not specified) (day 3 and 5)	Negative (n=2)	No (n=2)
La Cour Freiesleben et al. (140)	Denmark	13 cases	NI	NI	Pregnant (n=12)<br><br>Abortion (n=1)	NA	GA at diagnosis: first trimester	Abortion (n=1)	NI	NI	NA	NA	NA	NA	NA
Lang et al. (128)	6 Feb 2020<br><br>China	1 case	30	None	Caesarean	Emergency CS due to decrease in fetal heart rate	35^{+5}	Live birth	No	No	NI	NI	RT-PCR for cord blood, amniotic fluid, placenta; neonatal oropharyngeal swab (after birth); breastmilk; repeated neonatal	Negative	No

													oropharyngeal swabs		
Lee et al. (79)	6 Mar 2020<br><br>Korea	1 case	28	None	Caesarean	Cephalopelvic disproportion	37^{+6}	Live birth	No	No	3130	None	Placenta, amniotic fluid, cord blood and two neonatal nasopharyngeal swab	Negative	No
Li et al. (80)	6 – 24 Feb 2020<br><br>China	1 case	30	Fetal distress	Caesarean	Fetal distress	35^{+0}	Live birth	No	No	NI	NI	Placenta, amniotic fluid, cord blood, breast milk. Newborn´s oropharyngeal swab at birth; oropharyngeal swab, blood, feces and urine samples until 2 days after birth (7 different times)	Negative	No

Li et al. (170)<br><br>Cao et al. (17)	24 Jan - 29 Feb 2020<br><br>China	16 cases	Mean: 30.9±3.2	Chronic hypertension (n=1); polycystic ovary syndrome (n=1); gestational diabetes (n=3); PROM (n=1); gestational hypertension (n=3); hypothyroidism (n=2); preeclampsia (n=1); sinus tachycardia (n=1); fetal distress (n=2); COVID-19 Pneumonia (n=16)	Caesarean (n=14)<br><br>Vaginal (n=2)	COVID-19 Pneumonia (n=14); Emergency CS (n=12)	Mean: 38±0.2<br><br>Preterm birth (n=3)	Live births (n=17; 1 twin pregnancy)	No	No	Mean: 3066.7± 560.2 Low birth weight (n=3)	None	Neonatal throat swabs (one 4 days and another 14 days after birth) (n=3)	Negative (n=3)	No
Li et al. (170)	24 Jan - 29 Feb 2020<br><br>China	18 cases	Mean: 29.8±2.3	Gestational hypertension (n=2); fetal distress (n=1)	Caesarean (n=16)<br><br>Vaginal (n=2)	Emergency CS (n=10)	Mean: 38 ± 2.9<br><br>Preterm birth (n=3)	Live births (n=19; 1 twin pregnancy)	No	No	Mean: 3198.7± 522.6<br><br>Low birth weight (n=2)	None	NI	NI	No
Li et al. (99)	Published July 2020<br><br>China	1 case	31	COVID-19 pneumonia; ARDS; septic shock	Caesarean	Emergency CS	35^{+2}	Live birth	Yes<br><br>(mechanical ventilation, ECMO therapy)	No	2700	Severe asphyxia (mechanical ventilation)	NI	NI	Yes (2 hours after birth)
Liao et al. (81)	11 Feb 2020<br><br>China	1 case	25	Fetal distress	Caesarean	Fetal distress	GA at admission 35^{+3}	Live birth	No	No	NI	NI	Placenta, amniotic fluid, cord blood, neonatal serum, neonatal throat swab and	Negative	NI

													neonatal anal swab		
Liao et al. (159)	20 Jan – 2 Mar 2020<br><br>China	1 case	29	NI	Vaginal	NA	40^{+4}	Live birth	NI	No	NI (Mean of all 10 cases 3283 ± 449)	NI	Not tested	Not tested	No
Liao et al. (159)	20 Jan – 2 Mar 2020<br><br>China	1 case	36	NI	Vaginal	NA	39^{+0}	Live birth	NI	No	NI (Mean of all 10 cases 3283 ± 449)	NI	Not tested	Not tested	No
Liao et al. (159)	20 Jan – 2 Mar 2020<br><br>China	1 case	30	NI	Vaginal	NA	39^{+5}	Live birth	NI	No	NI (Mean of all 10 cases 3283 ± 449)	NI	Not tested	Not tested	No
Liao et al. (159)	20 Jan – 2 Mar 2020<br><br>China	1 case	36	NI	Vaginal	NA	37^{+6}	Live birth	NI	No	NI (Mean of all 10 cases 3283 ± 449)	NI	Neonatal throat swabs (x2, at least 24 hours apart)	Negative	No
Liao et al. (159)	20 Jan – 2 Mar 2020<br><br>China	1 case	36	NI	Vaginal	NA	38^{+1}	Live birth	NI	No	NI (Mean of all 10 cases 3283 ± 449)	NI	Neonatal throat swabs (x2, at least 24 hours apart)	Negative	No

Liao et al. (159)	20 Jan – 2 Mar 2020<br><br>China	1 case	30	NI	Vaginal	NA	40^{+1}	Live birth	NI	No	NI (Mean of all 10 cases 3283 ± 449)	NI	Neonatal throat swabs (x2, at least 24 hours apart)	Negative	No
Liao et al. (159)	20 Jan – 2 Mar 2020<br><br>China	1 case	33	NI	Vaginal	NA	39^{+4}	Live birth	NI	No	NI (Mean of all 10 cases 3283 ± 449)	NI	Neonatal throat swabs (x2, at least 24 hours apart)	Negative	No
Liao et al. (159)	20 Jan – 2 Mar 2020<br><br>China	1 case	33	NI	Vaginal	NA	36^{+2}	Live birth	NI	No	NI (Mean of all 10 cases 3283 ± 449)	NI	Neonatal throat swabs (x2, at least 24 hours apart)	Negative	No
Liao et al. (159)	20 Jan – 2 Mar 2020<br><br>China	1 case	27	NI	Vaginal	NA	40^{+2}	Live birth	NI	No	NI (Mean of all 10 cases 3283 ± 449)	NI	Neonatal throat swabs (x2, at least 24 hours apart)	Negative	No
Liao et al. (159)	20 Jan – 2 Mar 2020<br><br>China	1 case	29	NI	Vaginal	NA	37^{+2}	Live birth	NI	No	NI (Mean of all 10 cases 3283 ± 449)	Hyaline membrane disease	Neonatal throat swabs (x2, at least 24 hours apart)	Negative	No
Liu et al (64)	31 Jan – 29 Feb 2020<br><br>China	1 case	36	PROM; COVID-19 pneumonia	NI	NI	36^{+3}	Live birth	NI	No	2840	NICU admission	Neonatal throat swabs, gastric fluid, urine, feces; amniotic fluid and	Negative (all samples)	No

													umbilical cord blood		
Liu et al (64)	31 Jan – 29 Feb 2020<br><br>China	1 case	26	PROM; COVID-19 pneumonia	NI	NI	35^{+2}	Live birth	NI	No	2500	NICU admission	Neonatal throat swabs, gastric fluid, urine, feces; amniotic fluid and umbilical cord blood	Negative (all samples)	No
Liu et al (64)	31 Jan – 29 Feb 2020<br><br>China	1 case	38	COVID-19 pneumonia	NI	NI	38^{+2}	Live birth	NI	No	2920	NICU admission	Neonatal throat swabs, gastric fluid, urine, feces; amniotic fluid and umbilical cord blood; breastmilk after the first lactation	Negative (all samples)	No
Liu et al (64)	31 Jan – 29 Feb 2020<br><br>China	1 case	27	COVID-19 pneumonia	NI	NI	39^{+5}	Live birth	NI	No	3190	NICU admission	Neonatal throat swabs, gastric fluid, urine, feces; amniotic fluid and	Negative (all samples)	No

													umbilical cord blood; breastmilk after the first lactation		
Liu et al (64)	31 Jan – 29 Feb 2020<br><br>China	1 case	30	COVID-19 pneumonia	NI	NI	39^{+1}	Live birth	NI	No	3290	NICU admission	Neonatal throat swabs, gastric fluid, urine, feces; amniotic fluid and umbilical cord blood; breastmilk after the first lactation	Negative (all samples)	No
Liu et al (64)	31 Jan – 29 Feb 2020<br><br>China	1 case	26	COVID-19 pneumonia	NI	NI	37^{+5}	Live birth	NI	No	2640	NICU admission	Neonatal throat swabs, gastric fluid, urine, feces; amniotic fluid and umbilical cord blood; breastmilk after the first lactation	Negative (all samples)	No

Liu et al (64)	31 Jan – 29 Feb 2020<br><br>China	1 case	30	COVID-19 pneumonia	NI	NI	38^{+6}	Live birth	NI	No	3710	NICU admission	Neonatal throat swabs, gastric fluid, urine, feces; amniotic fluid and umbilical cord blood; breastmilk after the first lactation	Negative (all samples)	No
Liu et al (64)	31 Jan – 29 Feb 2020<br><br>China	1 case	27	COVID-19 pneumonia	NI	NI	41^{+2}	Live birth	NI	No	4120	NICU admission	Neonatal throat swabs, gastric fluid, urine, feces; amniotic fluid and umbilical cord blood; breastmilk after the first lactation	Negative (all samples)	No
Liu et al (64)	31 Jan – 29 Feb 2020<br><br>China	1 case	26	COVID-19 pneumonia	NI	NI	39^{+0}	Live birth	NI	No	3160	NICU admission	Neonatal throat swabs, gastric fluid, urine, feces; amniotic fluid and umbilical cord	Negative (all samples)	No

													blood; breastmilk after the first lactation		
Liu et al (64)	31 Jan – 29 Feb 2020<br><br>China	1 case	30	COVID-19 pneumonia	NI	NI	38^{+0}	Live birth	NI	No	3860	NICU admission	Neonatal throat swabs, gastric fluid, urine, feces; amniotic fluid and umbilical cord blood; breastmilk after the first lactation	Negative (all samples)	No
Liu et al (64)	31 Jan – 29 Feb 2020<br><br>China	1 case	33	COVID-19 pneumonia	NI	NI	39^{+0}	Live birth	NI	No	3930	NICU admission	Neonatal throat swabs, gastric fluid, urine, feces; amniotic fluid and umbilical cord blood; breastmilk after the first lactation	Negative (all samples)	No

Liu et al (64)	31 Jan – 29 Feb 2020<br><br>China	1 case	33	COVID-19 pneumonia	NI	NI	38^{+0}	Live birth	NI	No	3090	NICU	Neonatal throat swabs, gastric fluid, urine, feces; amniotic fluid and umbilical cord blood; breastmilk after the first lactation	Negative (all samples)	No
Liu et al. (27)	20 Jan-10 Feb 2020<br><br>China	11 cases	23-40 Mean: 32±5	Thalassemia and gestational diabetes (n=1); previous mitral and tricuspid valves replacement (n=1); placenta previa (n=1); COVID-19 Pneumonia (n=15)	Caesarean (n=10)<br><br>Vaginal (n=1)	NI	GA at admission: 34-38 (Mean: 37)<br><br>Preterm deliveries (n=3)	Live births (n=11)	No	No	NI	None	RT-PCR in neonatal sample (not specified)	Negative	No
Liu et al. (27)<br><br>Li et al. (48)	20 Jan-10 Feb 2020<br><br>China	4 cases	29-40 Mean: 34±5	COVID-19 Pneumonia (n=4)	Pregnant (n=4)	NA	GA at admission: 12-32 (3 in the second trimester and 1 in the third trimester)	NA	No	No	NA	NA	NA	NA	NA

Liu et al. (28) Luo et al. (29) Wang et al. (82) Yu et al. (75)	1 Jan - 18 Feb 2020 China	1 case	34	Hypothyroidism	Caesarean	NI	39^{+6}	Live birth	No	No	3250	Mild shortness of breath symptoms. mild pulmonary infection on Chest x-ray	Placenta, cord blood, breast milk, neonatal throat swab (36h after birth) and two weeks after birth	Positive (neonatal throat swab 36 h after birth) and negatives (two weeks after birth)	No
Liu et al. (28) Luo et al. (29) Yu et al. (75)	1 Jan - 8 Feb 2020 China	1 case	31	None	Caesarean	NI	41^{+2}	Live birth	No	No	3200	None	Neonatal throat swab	Negative	No
Liu et al. (28) Luo et al. (29) Yu et al. (75)	1 Jan - 8 Feb 2020 China	1 case	34	Uterine scarring	Caesarean	NI	38^{+4}	Live birth	No	No	3250	None	Neonatal throat swab	Negative	No
Liu et al. (160)	27 Jan- 14 Feb 2020 China	41 cases	Median:30 (22- 42)	Gestational diabetes (n=4); gestational hypertension (n=3); hepatitis B infection (n=1)	Pregnant (n=22); deliveries (n=19), type of delivery not informed	NI	22^{+0} - 40^{+5}	Live births (n=41)	No	No	NI	None	NI	NI	No

Liu et al. (161)	20 Jan - 20 Mar 2020<br><br>China	51 cases	31.94±4.02	NI	Caesarean (n=48)<br><br>Vaginal (n=3)	NI	Median: 38 (range: 35^{+1} to 41^{+2})<br><br>Preterm birth (n=6)	Live births (n=51)	NI (without mechanical ventilation n=51)	No (n=51)	Median: 3080 (range: 1990 - 3950)	NICU admission (n=51);<br><br>No mechanical ventilation (n=51);<br><br>Transient sign of abdominal distension or vomiting (n=10); NEC (n=1)	Neonatal pharyngeal swabs on days 0, 1, and 5 after birth; neonatal blood (n=51)	Negative (n=51)<br><br>Non-reactive IgG (n=51) Slightly higher IgM (n=10)	No (n=51)
Lokken et al. (49)	21 Jan – 17 Apr 2020<br><br>USA	46 cases	Median: 29 (IQR=26-34)	Overweight (n=12); obese (n=15); asthma (n=4); type 2 diabetes (n=3); hypothyroidism (n=3); hypertension (n=2); Crohn's disease with immunosuppressive therapy (n=1); heart valve repair (n=1); papillary thyroid carcinoma with thyroidectomy (n=1); and seizure disorder (n=2); Asymmetrical IUGR (n=1); gestational diabetes (n=1); gestational hypertension (n=2); cholestasis	Caesarean (n=3)<br><br>Vaginal (n=5)<br><br>Pregnant (n=38)	To improve maternal respiratory status due to COVID 19 disease (n=2)<br><br>NI (n=1)	GA at symptom onset (n=46): 27.0 (IQR: 21.0-33.9)<br><br>GA at delivery (n=8): 38.4 (IQR: 37.5-39.8)<br><br>Preterm birth (n=1/8)<br><br>Stillbirth (n=1/8): 38.7 weeks	Live birth (n=7)<br><br>Stillbirth (n=1)<br><br>Pregnant (n=38)	Yes (n=1)<br><br>Nasal cannula (n=4); high-flow nasal cannula (n=1)	No	NI	NI	Live births: NI<br><br>Stillbirth: PCR testing of placental and fetal tissues	Live births: NI<br><br>Stillbirth: Negative	No (n=7)

				(n=1); placenta abruption (n=1); fetal distress (n=3)											
London et al. (141)<br><br>Blitz et al. (35)	15 Mar - 10 Apr 2020<br><br>USA	68 cases	Median: 30 (IQR: 25.0-34.4)	Gestational diabetes (n=7/68); diabetes (n=2/68); chronic hypertension (n=2/68); asthma (n=2/68); cholestasis of pregnancy (n=2/68); preeclampsia (n=4/55)	: Caesarean (n=22)<br><br>Vaginal (n=33)<br><br>Fetal demise (n=1)<br><br>Pregnant (n=12)	NI	Preterm birth (n=9/55)<br><br>Fetal demise at 17 weeks (n=1/68)	Live births (n=55/68)<br><br>Fetal demise at 17 weeks (n=1/68)<br><br>Pregnant (n=12/68)	NI<br><br>Required any respiratory support (n=12/68); mechanical ventilation (n=1/68)	No (n=68)	NI	NI	Nasopharyngeal swabs on day 0 of life	Negative (n=48)	NI
Lowe et al. (100)	Published 11 April 2020<br><br>Australia	1 case	31	NI	Vaginal	NA	40^{+3}	Live birth	No	No	NI	None	RT-PCR in neonatal sample (not specified) (24 hours)	Negative	No
Lu et al. (101)	11 Feb 2020<br><br>China	1 case	22	None	Caesarean	Emergency CS (irregular contractions)	38	Live birth	No	No	3470	None	Neonatal nasopharyngeal swabs (at birth, 24 hours after birth, 1 month); oropharyngeal swabs (at birth, 24 hours after birth); blood (at birth, 24 hours after birth);	Negative	No

													stool swab (day 6 after birth)		
Lucarelli et al (65)	Published on May 28 2020<br><br>USA	1 case	38	COVID-19 pneumonia	Pregnant	NA	GA at admission 28^{+0}	NA	Yes (mechanical ventilation)	No	NA	NA	NA	NA	NA
Lucarelli et al (65)	Published on May 28 2020<br><br>USA	1 case	26	COVID-19 pneumonia	Pregnant	NA	GA at admission 29^{+6}	NA	Yes (mechanical ventilation)	No	NA	NA	NA	NA	NA
Lucarelli et al (65)	Published on May 28 2020<br><br>USA	1 case	46	COVID-19 pneumonia; in vitro fertilization with donor egg	Pregnant	NA	GA at admission 23^{+5}	NA	Yes (mechanical ventilation)	No	NA	NA	NA	NA	NA
Luo et al. (142)	1 Feb - 15 Mar 2020<br><br>China	23 cases	Mean: 29.2±4.9 (Range: 21-40)	COVID-19 pneumonia (n=9)	Caesarean (n=17)<br><br>Vaginal (n=6)	NI	Third trimester (n=23)<br><br>Preterm (n=3/23)	Live birth (n=23)	No (without mechanical ventilation)	No	NI	NICU admission (n=6)	RT-PCR of neonatal throat swabs (after delivery) (n=21); breastmilk (until one week postpartum; 1 sample in the 12th day after birth; 1 sample in the 15th	Negative neonatal throat swabs (n=21)<br><br>Negative breastmilk samples (n=23)<br><br>Non-reactive IgG & IgM in neonatal serum (n=8)	No (n=23)

													day after birth) (n=23); neonatal serum IgG & IgM test (one month after birth) (n=8); IgG & IgM test in breastmilk (n=7)	Reactive IgM in breastmilk (n=5)<br><br>Non-reactive IgG in breastmilk (n=7)	
Luo et al. (29)<br><br>Zhu et al. (32)	20 Jan - 5 Feb 2020<br><br>China	1 case	25	Fetal distress, oligohydramnios	Caesarean	NI	38^{+4}	Live birth	NI	No	2450	SGA, vomiting, feeding intolerance and mild bloating	Placenta, neonatal throat swab sample (day 3 after birth)	Negative	No
Luo et al. (29)<br><br>Zhu et al. (32)	20 Jan - 5 Feb 2020<br><br>China	1 case	35	Scarred uterus, PROM	Caesarean	NI	33^{+6}	Live birth	NI	No	2050	Shortness of breath	Placenta, neonatal throat swab sample (day 1 after birth)	Negative	No
Luo et al. (29)<br><br>Zhu et al. (32)	20 Jan - 5 Feb 2020<br><br>China	1 case	35	Fetal distress, PROM	Vaginal	NA	34^{+2}	Live birth	NI	No	2350	Shortness of breath	Placenta, neonatal throat swab sample (day 1 after birth)	Negative	No

Luo et al. (29)<br><br>Zhu et al. (32)	20 Jan - 5 Feb 2020<br><br>China	1 case	30	Fetal distress, vaginal bleeding in the third trimester	Caesarean	NI	34^{+5}	Live birth	NI	No	2200	Increased heart rate, refractory shock, gastric bleeding, multiple organ failure and disseminated intravascular coagulation	Placenta, neonatal throat swab sample (day 9 after birth)	Negative	Yes
Luo et al. (29)<br><br>Zhu et al. (32)	20 Jan - 5 Feb 2020<br><br>China	1 case	30	Cholecystitis and fever	Caesarean	NI	39^{+0}	Live birth	NI	No	3030	Diffusely scattered rashes throughout the body, edema and facial skin lesions	Placenta	Negative	No
Luo et al. (29)<br><br>Zhu et al. (32)	20 Jan - 5 Feb 2020<br><br>China	1 case	30	Fetal distress, placenta previa, Polyhydramnios	Caesarean	NI	37^{+0}	Live birth	NI	No	3800	LGA	Placenta, neonatal throat swab sample (day 1 after birth)	Negative	No (still hospitalized)
Luo et al. (29)<br><br>Zhu et al. (32)	20 Jan - 5 Feb 2020<br><br>China	1 case	30	Fetal distress	Caesarean	NI	34^{+6}	Live birth	NI	No	2300	Shortness of breath, fever, gastrointestinal hemorrhage, disseminated intravascular coagulation	Placenta, neonatal throat swab sample (day 7 after birth)	Negative	No
Luo et al. (29)<br><br>Zhu et al. (32)	20 Jan - 5 Feb 2020<br><br>China	1 case	29	Fetal distress, PROM	Vaginal	NA	31^{+0}	Live birth (n=2; twin pregnancy)	NI	No	1520/ 1720	Shortness of breath, cyanosis	Placenta, neonatal throat swab sample (day 1 after birth)	Negative	No (still hospitalized)

Luo et al. (29) Zhu et al. (32)	20 Jan - 5 Feb 2020 China	1 case	34	None	Caesarean	NI	39^{+0}	Live birth	NI	No	2810	SGA, moaning, shortness of breath, cyanosis, fever, tachypnea, cyanosis, fever and refusing milk	Placenta, neonatal throat swab sample (day 1 after birth)	Negative	No (still hospitalized)
Luo et al. (29) Zhu et al. (32)	1 Jan - 8 Feb 2020 China	1 case	30	Polycystic ovary syndrome	Caesarean	NI	38^{+5}	Live birth	No	No	3350	None	Not tested	Not tested	No
Luo et al. (29) Zhu et al. (32)	1 Jan - 8 Feb 2020 China	1 case	33	Uterine scarring	Caesarean	NI	37^{+0}	Live birth	No	No	3000	None	Not tested	Not tested	No
Luo et al. (29) Yu et al. (75)	1 Jan - 8 Feb 2020 China	1 case	29	None	Caesarean	NI	40^{+4}	Live birth	No	No	3500	None	Not tested	Not tested	No
Luo et al. (29) Yu et al. (75)	1 Jan - 8 Feb 2020 China	1 case	34	Uterine scarring	Caesarean	NI	38^{+2}	Live birth	No	No	3300	None	Not tested	Not tested	No
Lumbreras et al. (50)	Until May 17 2020 Mexico	301 cases	Median: 30 (IQR 26-33)	Diabetes (n=11); Obesity (n=45); Asthma (n=8); Hypertension (n=14); Chronic obstructive pulmonary	NI	NI	NI	NI	Yes (n=8) Mechanical ventilation (n=3)	No (n=301)	NI	NI	NI	NI	NI

				disease (n=2);<br>Chronic kidney disease (n=2);<br>COVID-19 Pneumonia (n=32)											
Lumbreras et al. (50)	Until May 17 2020<br><br>Mexico	7 cases	37 (IQR 26- 39)	Diabetes (n=4);<br>Obesity (n=2);<br>COVID-19 Pneumonia (n=6)	NI	NI	NI	NI	Yes (n=2)<br><br>Mechanical ventilation (n=1)	Yes (n=7)	NI	NI	NI	NI	NI
Lyra et al. (102)	Published on 1 Jun 2020<br><br>Portugal	1 case	35	None	Caesarean	Bishop score < 4 and prior history of a CS	39^{+6}	Live birth	No	No	3110	None	Placenta, amniotic fluid, colostrum;<br><br>neonatal nasal and oropharyngeal swabs (0h, 48h and 7 days after birth)	Results not available for placenta, amniotic fluid and colostrum<br><br>Negative (neonatal swabs)	No
Martínez-Perez et al. (143)	12 Mar - 6 Apr 2020<br><br>Spain	82 cases	*Asymptomatic/mild COVID-19*<br>Vaginal delivery (n=41), Median: 35 (19-43)<br><br>Caesarean delivery (n=37), Median: 33 (19-48)	Obesity (n=19); asthma (n=6); PROM (n=18); gestational diabetes (n=1); preeclampsia (n=4); hypothyroidism (n=5); epilepsy (n=1); subek muscular dystrophy (n=1); myopathy (n=1), heterozygous factor V mutation (n=1); psychiatric	*Total sample:*<br>Caesarean (n=41/82); Vaginal (n=41/82)<br><br>*Asymptomatic/mild COVID-19:*<br>Caesarean (n=37/78); Vaginal (n=41/78)	*Asymptomatic/mild COVID-19*: obstetrical indications (n=29/37); COVID19 symptoms (n= 8/37); in labor CS (n=24/37); spontaneous onset of	*Asymptomatic/mild COVID-19*<br>Vaginal delivery: Median 39^{+1} (27^{+3}-41^{+3});<br>Caesarean delivery: Median 38^{+3} (25^{+0}-41^{+4});<br><br>*Severe COVID-19 symptoms*<br>Median 29^{+6} (28^{+0}-34^{+0})	Live birth (n=82)	Yes (n=9/82): Asymptomatic/mild COVID-19 (n=5/78); severe COVID-19 symptoms (n=4/4)<br><br>Mechanical ventilation (n=6/82);	No (n=82)	*Asymptomatic/mild COVID-19*<br>Vaginal delivery (n=41), Median: 3060 (940-4750);<br>Caesarean delivery (n=37), Median:	NICU admission (n=22/82):<br><br>*Asymptomatic/mild COVID-19*<br>Vaginal delivery (n=8/41);<br>Caesarean delivery (n=11/37)<br><br>*Severe COVID-19*	Neonatal nasopharyngeal swab (at birth and 48h after birth) (n=72)	Positive at birth (n=3/72)<br><br>Negative (n=3/3, 48h after birth)<br><br>Positive (n=2/2, 48h after birth) (2 newborns with	No (n=82)

			Severe COVID-19 (n=4) Median: 36 (22-47)	disorders (n=4); unspecified autoimmune disease (n=1); hyperprolactinemia (n=1); gastritis (n=1); vitiligo (n=1); chronic hepatitis C infection (n=1); homocysteine mutation (n=1); anti-Kell alloimmunization (n=1); concomitant pyelonephritis (n=1); myomatosis (n=1), mutation of methylenetetrahydrofolate reductase (n=1); ischemic cardiomyopathy (n=1)	*Severe COVID-19 symptoms:* Caesarean (n=4/4)	labor (n=16/24)<br><br>*Severe COVID-19:* prelabor CS (n=4/4)	Preterm birth (n=25/82) [Iatrogenic preterm birth, n=12/25]		oxygen supplementation at admission (n=15/82)		3210 (910-4510)<br><br>*Severe COVID-19 symptoms* (n=4) Median: 1450 (1110-1580)	*symptoms* (n=3/4)		negative test at birth which developed symptoms within 10 days)	
Mehta et al. (107)	Published at May 16 2020<br><br>USA	1 case	39	In-vitro fertilization; COVID-19 pneumonia; ARDS	Caesarean	Emergency CS due to rapid maternal decompensation, ARDS	28^{+0}	Live birth (n=2, twin pregnancy)	Yes<br><br>(endotracheal intubation, mechanical ventilation)	No	T1: 925<br><br>T2: 1050	NICU admission (n=2)<br><br>T1: No infection symptoms<br><br>T2: Required invasive mechanical ventilation for alveolar hemorrhage	Neonatal RT- PCR at 72 after birth	T1: Positive<br><br>T2: Negative	No (n=2)

Mendonza et al. (144)	13 Mar - 10 Apr 2020<br><br>Spain	42 cases	*Total cases (n=42)* Median: 32 (IQR: 26-37.5)<br><br>*Non-severe cases (n=34)* Median: 30.9 (IQR: 25-41.8)<br><br>*Severe cases (n=8)* Median: 39.4 (IQR: 34.2-44.5)	Severe COVID-19 pneumonia (n=8); preeclampsia and/or HELLP syndrome (n=6, all in women with severe COVID); ART (n=2); pre-pregnancy diabetes (n=1)	*Non-severe cases (n=34):* NI<br><br>*Severe cases (n=8):* Caesarean (n=4/8); Pregnant (n=4/8)	COVID-19 worsening in (n=3/4); HELLP syndrome (n=1/4)	*Total cases (n=42)* Median: 31^{+6} (25.9-36.1)<br><br>*Non-severe cases (n=34)* Median: 32^{+8} (26.7-36.1)<br><br>*Severe cases (n=8)* Median: 28^{+6} (22.3-32.4)	NI	Yes (n=8/42)	No (n=42)	NI	NI	NI	NI	NI
Mohr-Sasson et al. (145)	Mar- Apr 2020<br><br>Israel	11 cases	Mean: 28 (Range: 24-35)	None	Caesarean (n=2)<br><br>Vaginal (n=9)	COVID-19 (n=1); non-reassuring fetal monitor (n=1)	Third trimester (n=11)	NI	No (n=11; 1 was intubated)	No	NI	NI	NI	NI	NI
Mulvey et al. (66)	Published on 25 Apr 2020<br><br>USA	1 case	35	Placenta focal accrete	Vaginal	NA	39	Live birth	No	No	NI	NI	NI	NI	No
Mulvey et al. (66)	Published on 25 Apr 2020<br><br>USA	1 case	30	Pelvic fracture	Vaginal	NA	38	Live birth	No	No	NI	NI	NI	NI	No

Mulvey et al. (66)	Published on 25 Apr 2020<br><br>USA	1 case	29	Polycystic ovary syndrome; iron deficiency anemia	Vaginal	NA	40	Live birth	No	No	NI	NI	NI	NI	No
Mulvey et al. (66)	Published on 25 Apr 2020<br><br>USA	1 case	40	Hypothyroidism	Caesarean	History of CS	39	Live birth	No	No	NI	NI	NI	NI	No
Mulvey et al. (66)	Published on 25 Apr 2020<br><br>USA	1 case	26	History of intrauterine fetal demise	Vaginal	NA	38	Live birth	No	No	NI	NI	NI	NI	No
Nesr et al. (103)	Published on<br><br>USA	1 case	34	Trombocytopenia (auto-imune disease); bleeding	Pregnant	NA	GA at admission 20	NA	No	No	NA	NA	NA	NA	NA
Nie et al. (67)	Jan - Feb 2020<br><br>China	33 cases	Mean 30.5 (SD= 3.1) (range24-36)	PROM (n=3); pregnancy hypertensive diseases (n=2); gestational diabetes (n=2); fetal distress (n=4); cardiovascular and cerebrovascular diseases (n=9); digestive system disease (n=1); endocrine system disease (n=2); nervous system disease (n=1); infectious disease (n=1); depressive disorder (n=1);	Caesarean (n=22)<br><br>Vaginal (n=5)<br><br>Induced abortion (for personal reasons) (n=1)<br><br>Pregnant (n=5)	NI	Second trimester (n=3/33, 17w, 20w, and 26w)<br><br>Third trimester (n=30/33)<br><br>Preterm birth (n=10/28)	Live birth (n=28; 1 twin pregnancy)	No (n=33)<br><br>Non-invasive mechanical ventilation (n=1); oxygen supplementati on by nasal cannula or mask (n=29)	No (n=33)	Mean 2988 (SD= 502)	NICU admission (n=1)<br><br>ARDS (n=1)	Throat swabs (n=26); Cord blood and placental samples (n=1, only for the positive case in throat swab).	Negative throat swabs (n=25)<br><br>Positive throat swabs (n=1)<br><br>Negative (Cord blood and placental samples) (n=1)<br><br>Positive neonatal throat sample was	No (n=28)

				COVID-19 pneumonia (n=27)										negative on days 4, 8, and 15 after birth	
Ochiai et al. (162)	6-27 Apr 2020 Japan	1 case	33	None	Caesarean	Elective CS	39^{+4}	Live Birth	No	No	3715	None	RT-PCR in neonatal sample (not specified)	Negative	No
Ochiai et al. (162)	6-27 Apr 2020 Japan	1 case	32	None	Caesarean	Elective CS	37^{+2}	Live Birth	No	No	2805	None	RT-PCR in neonatal sample (not specified)	Negative	No
Panichaya et al. (129)	Published on May 2020 Thailand	1 case	43	Fetal down syndrome	Vaginal	NA	21	Termination of pregnancy (Down syndrome)	No	No	NA	NA	RT-PCR from placental swab, fetal nasopharyngeal and throat swab	Negative	NA
Patanè et al. (51)	5 Mar-21 Apr 2020 Italy	20 cases	NI	NI	NI	NI	Third trimester (n=20)	Live birth (n=20)	NI	NI	NI	NI	Placenta, Neonatal nasopharyngeal swab at birth, 24h and 7 days of life	Negative (n=20)	NI
Patanè et al. (51)	5 Mar-21 Apr 2020 Italy	1 case	NI	None	Vaginal	NA	37^{+6}	Live Birth	NI	NI	2660	Mild initial feeding difficulties; remained asymptomatic.	Placenta, Newborn´s nasopharyngeal swab at birth, 24h	Positive (fetal side placenta, swabs at birth, 24h and 7 days)	No

													and 7 days of life.		
Patanè et al. (51)	5 Mar-21 Apr 2020<br><br>Italy	1 case	NI	None	Caesarean	Non reassuring fetal status	35^{+1}	Live Birth	NI	NI	2686	NICU admission<br><br>Feeding difficulties in the first days of life.	Placenta, nasopharyngeal swab at birth and 7 days of life	Negative (swab at birth); Positive (fetal side placenta, swab 7 days of life)	No
Peng et al. (104)	Published on May 2020<br><br>China	1 case	25	COVID-19 Pneumonia; Fetal distress	Caesarean	Fetal intrauterine distress	35^{+3}	Live birth	No<br><br>Oxygen supplement	No	2600	NICU admission<br><br>Tachypnea, moaning and periodic breath immediately after birth	Vaginal secretion; placenta; cord blood; amniotic fluid.<br><br>Newborn: throat swab, anal swab, serum, and urine samples tested at 2 hours, 1day, 2 days, 3 days, 7 days, 14 days; sputum tested at 1 day and 7 days; breast milk tested at	Negative (all samples)	No

													day 2, 3, 4, 5, 6, 7, 10, 14		
Penfield et al. (68)	1 Mar - 20 Apr 2020<br><br>USA	1 case	37	COVID 19 status critical	Caesarean	NI	36^{+6}	Live birth	NI	NI	NI	NI	PCR of membrane sample (30 minutes after delivery); PCR nasopharyngeal swabs (day 2, day 4)	Positive: Membrane sample<br><br>Negative: Nasopharyngeal swabs	NI
Penfield et al. (68)	1 Mar - 20 Apr 2020<br><br>USA	1 case	36	COVID 19 status critical	Caesarean	NI	26^{+5}	Live birth	NI	NI	NI	NI	PCR of membrane sample (30 minutes after delivery); PCR nasopharyngeal swabs (day 1, day 5)	Positive: Membrane sample<br><br>Negative: Nasopharyngeal swabs	NI
Penfield et al. (68)	1 Mar - 20 Apr, 2020<br><br>USA	1 case	38	COVID 19 status critical	Caesarean	NI	38^{+3}	Live birth	NI	NI	NI	NI	PCR of membrane sample (30 minutes after delivery); PCR nasopharyngeal swabs	Negative: Membrane sample and nasopharyngeal swabs	NI

													(day 1, day 3)		
Penfield et al. (68)	1 Mar - 20 Apr 2020<br><br>USA	1 case	40	COVID 19 status Severe	Caesarean	NI	34^{+2}	Live birth	NI	NI	NI	NI	PCR of placental sample (30 minutes after delivery); PCR nasopharyngeal swabs (day 1, day 4, day 5)	Positive: Placental sample<br><br>Negative: Nasopharyngeal swabs	NI
Penfield et al. (68)	1 Mar - 20 Apr, 2020<br><br>USA	1 case	26	COVID 19 status Severe	Vaginal	NA	37^{+6}	Live birth	NI	NI	NI	NI	PCR of membrane sample (30 minutes after delivery); PCR nasopharyngeal swabs (day 1, day 3)	Negative: Membrane sample and nasopharyngeal swabs	NI
Penfield et al. (68)	1 Mar - 20 Apr, 2020<br><br>USA	1 case	34	NI	Vaginal	NA	37^{+1}	Live birth	NI	NI	NI	NI	PCR of membrane sample (30 minutes after delivery); PCR nasopharyngeal swabs	Negative: Membrane sample and nasopharyngeal swabs	NI

													(day 3, day 4)		
Penfield et al. (68)	1 Mar - 20 Apr 2020 USA	1 case	23	NI	Vaginal	NA	41^{+3}	Live birth	NI	NI	NI	NI	PCR of membrane sample (30 minutes after delivery); PCR nasopharyngeal swabs (day 2)	Negative: Membrane sample and nasopharyngeal swabs	NI
Penfield et al. (68)	1 Mar - 20 Apr 2020 USA	1 case	23	NI	Vaginal	NA	40^{+5}	Live birth	NI	NI	NI	NI	PCR of membrane sample (30 minutes after delivery); PCR nasopharyngeal swabs (day 2)	Negative: Membrane sample and nasopharyngeal swabs	NI
Penfield et al. (68)	1 Mar - 20 Apr 2020 USA	1 case	35	NI	Vaginal	NA	39^{+6}	Live birth	NI	NI	NI	NI	PCR of membrane sample (30 minutes after delivery); PCR nasopharyngeal swabs (day 1)	Negative: Membrane sample and nasopharyngeal swabs	NI

Penfield et al. (68)	1 Mar - 20 Apr, 2020<br><br>USA	1 case	34	NI	Vaginal	NA	40^{+0}	Live birth	NI	NI	NI	NI	PCR of membrane sample (30 minutes after delivery); PCR nasopharyngeal swabs (day 1)	Negative: Membrane sample and nasopharyngeal swabs	NI
Penfield et al. (68)	1 Mar - 20 Apr 2020<br><br>USA	1 case	22	NI	Vaginal	NA	41^{+0}	Live birth	NI	NI	NI	NI	PCR of membrane sample (30 minutes after delivery); PCR nasopharyngeal swabs (day 2)	Negative: Membrane sample and nasopharyngeal swabs	NI
Pereira et al. (69)	14 Mar-14 Apr 2020<br><br>Spain	60 cases	Median: 34 (range: 22-43)	Preeclampsia (n=3), fetal growth restriction (n=3), risk of preterm birth (n=3), deep venous thrombosis (n=2); HELLP syndrome (n=1); COVID-19 pneumonia (n=18)	Caesarean (n=5/23)<br><br>Spontaneous vaginal delivery (n=14/23)<br><br>Instrumental delivery (n=4/23)<br><br>Pregnant (n=37)	Maternal respiratory failure + uterine activity + beech presentation (n=1); non-progression of labor (n=2), induction failure (n=1); HELLP syndrome (n=1)	GA at COVID diagnosis: 32 weeks (range: 5-41).<br><br>1st trimester (1-12 w) (n=10); 2nd trimester (13-26 w) (n=16); 3rd trimester (27-41 w) (n=34)	Live birth (n=23)	Yes (n=1/60)<br><br>Oxygen support (n=10)	No (n=60)	NI	NICU admission (n=2)<br><br>Respiratory distress syndrome (n=1); hemolytic anemia (n=1)	Neonatal nasopharyngeal swab 2h after delivery (repeated after 24h if neonate presented respiratory distress syndrome);<br><br>RT-PCR of placental	Negative (neonatal swabs and placental samples)	NI

							Preterm birth (n=2/23)						samples (n=6/23)		
Perrone et al. (52)	Mar-Apr 2020<br><br>Italy	1 case	36	Hypothyroidism	Vaginal	NA	40^{+4}	Live birth	No	No	3790	None	Neonatal nasopharyngeal swab in the first day of life	Negative	No
Perrone et al. (52)	Mar-Apr 2020<br><br>Italy	1 case	35	Hypothyroidism	Vaginal	NA	38^{+5}	Live birth	No	No	3535	None	Placenta swab	Negative	No
Perrone et al. (52)	Mar-Apr 2020<br><br>Italy	1 case	32	Gestational diabetes, ARDS, COVID-19 pneumonia	Caesarean	Elective CS (maternal request)	38^{+2}	Live birth	No<br><br>Non-invasive ventilation (CPAP)	No	2290	None	Neonatal nasopharyngeal swab in the first day of life	Negative	No
Perrone et al. (52)	Mar-Apr 2020<br><br>Italy	1 case	26	Interstitial pneumonia suggestive of SARS-CoV-2 (RT-PCR negative)	Vaginal	NA	39^{+6}	Live birth	No	No	3720	None	Neonatal nasopharyngeal swab in the first day of life	Negative	No
Pierce-Williams et al. (146)	5 Mar-20 Apr 2020<br><br>USA	64 cases	Mean: 33.2±5.8	oligohydramnios (n=1); Preeclampsia/ gestational hypertension (n=2); presumed chorioamniotitis/ endometritis (n=3); PROM (n=1); Cardiac disease (including chronic	Vaginal (n=8)<br><br>Caesarean (n=24)<br><br>Pregnant (n=32)	Maternal status (n=8/15) (60%); Obstetric indication (n= 6/15) (40%)	Mean: 34.5±4.2<br><br>Preterm birth <37 weeks (n=19)<br><br>Preterm birth <34 weeks (n=10)	Live birth (n=33; 1 twin pregnancy)	NI (intubation (n=19))	No	Mean: 2403.3± 858.0	NICU admission (n=21)	RT-PCR in neonatal sample (not specified)	Negative (n=32)<br><br>Positive (48h after birth) (n=1)	No

				hypertension, cardiomyopathy) (n=11), pulmonary pathology (OSA, asthma, COPD) (n=16); intrauterine growth restriction (n=2)											
Piersigilli et al. (105)	1 Mar 2020<br><br>Belgium	1 case	NI	Preeclampsia, suspected cholecystitis, HELLP syndrome	Caesarean	HELLP syndrome; no signs of preterm labor	26^{+4}	Live birth	NI	No	960	NICU admission<br><br>Non-invasive ventilation; pneumothorax requiring drainage; hemodynamically significant patent ductus arteriosus; COVID-19 pneumonia	RT-PCR neonatal nasopharyngeal swab and breast milk	Positive: Neonatal swab 7 and 14 days after birth<br><br>Negative: Neonatal swab 28 days after birth and breastmilk	No
Polónia-Valente et al. (108)	17 Mar 2020<br><br>Portugal	1 case	31	None	Vaginal (vacuum extraction)	NA	38^{+0}	Live birth	No	No	3240	None	Neonatal nasal and oropharyngeal swabs (2h and 48h after birth)	Negative	No
Qadri et al. (163)	26 Mar - 10 Apr 2020<br><br>USA	16 cases	Range: 20-40	Obesity (n=10); PROM (n=2); COVID-19 pneumonia (n=4)	Caesarean (n=4)<br><br>Vaginal (n=8)<br><br>Pregnant (n=4)	Obstetrical indication (n=2), breech presentation (n=1), previous CS (n=1)	GA at hospital admission: $22\text{-}40^{+3}$<br><br>Preterm birth (spontaneous at 22 weeks) (n=1)	Live birth (n=12)	No (n=16)<br><br>Oxygen by nasal cannula (n=2)	No (n=16)	Range: 2830 - 4215	Neonate readmitted on day 4 due to hyperbilirubinemia (n=1)	RT-PCR in neonatal sample (not specified) 48h of life (n=16)	Negative (n=16)	No (n=16)

Qiancheng et al. (164)	15 Jan - 15 Mar 2020<br><br>China	28 cases	Median: 30 (26.75-32)	Hypertension (n=1); diabetes (n=2); chronic hepatitis B virus infection (n=2); hypothyroidism (n=1); COVID-19 pneumonia (n=26)	Caesarean (n=17)<br><br>Vaginal (n=5)<br><br>Medical termination of pregnancy (n=4)<br><br>Pregnant (n=2)	NI	GA at admission Median: 38 (36.5–39)<br><br>1st trimester (n=3), 2nd trimester (n=1), and 3rd trimester (n=24)<br><br>Preterm birth (n=1/22)	Live birth (n=23, 1 twin pregnancy)<br><br>Medical termination of pregnancy (n=4)	NI	No	Median: 3130 (IQR: 2915–3390)<br><br>Low birthweight (n=1/23)	None (n=23)	RT-PCR neonatal throat swab (24–48 hours)	Negative (n=23)	No (n=23)
Rabice et al. (130)	Published on May 2020<br><br>USA	1 case	36	Type 1 diabetes mellitus, mild intermittent asthma, obesity (pre-pregnancy BMI of 44 kg/m2), preeclampsia in two previous pregnancies and in the current pregnancy; acute pancreatitis	Caesarean	Repeat CS and preeclampsia	38^{+2}	Live birth	No<br><br>Oxygen via nasal cannula	No	NI	NI	NI	NI	No
Romero et al. (119)	Published 17 April 2020<br><br>Spain	1 case	44	COVID-19 pneumonia	Caesarean	Clinical condition (maternal respiratory deterioration)	GA at admission: 29^{+2}	Live birth	Yes<br><br>(intubation and mechanical ventilation)	No	NI	None	RT-PCR neonatal sample (non-specified)	Negative	No

Rosen et al. (131)	Published on 12 May 2020<br><br>USA	1 case	26	Acute severe ulcerative colitis; vaginal bleeding	Spontaneous abortion	NA	First-trimester (Very early pregnancy - GA not determined)	Spontaneous abortion	NI	No	NA	NA	NI	NI	NA
San-Juan et al. (153)	5 Mar-5 Apr 2020<br><br>Spain	20 cases	Mean: 31.4±6	Asthma (n=1); Hypertension (n=1)	NI	NI	GA at diagnosis: Mean 28.6±8	NI	NI	NI	NI	NI	NI	NI	NI
San-Juan et al. (153)	5 Mar-5 Apr 2020<br><br>Spain	32 cases	Mean: 32±7	Asthma (n=4); obesity (n=1); multiple sclerosis (n=1); gestational diabetes (n=2); COVID-19 pneumonia (n=32); ARDS=8	Caesarean (n=5/6)<br><br>Vaginal (n=1/6)	Urgent CS due to COVID-19 (n=3); NI (n=3)	GA at diagnosis: Mean 28.7±7.4 (IQR: 25-34)<br><br>Gestational trimester: 1st (n=1); 2nd (n=9); 3rd (n=22).<br><br>Preterm birth (n=2)	Live birth (n=6)<br><br>Pregnant (n=26)	Yes (n=2)<br><br>Invasive mechanical ventilation (n=2)	No (n=32)	Range: 1230-4000<br><br>Low birth weight (n=2)	1 admission to NICU due to preterm-derived complications (hyaline membrane disease, intraventricular hemorrhage grade 1, persistent arteriosus ductus without clinical repercussion)	RT-PCR in neonatal nasopharyngeal swabs	Negative (n=6)	No (n=5);<br><br>Still hospitalized (n=1)
Savasi et al. (147)	23 Feb-28 Mar 2020<br><br>Italy	77 cases	Median: 32 (range: 15–48)	Obesity (n=7), chronic hypertension (n=1), and advanced maternal age (older than 40 years, n=1); COVID-19 pneumonia (n=34)	Caesarean (n=22)<br><br>Vaginal (n=34)<br><br>NI (n=1)<br><br>Pregnant (n=20)	Urgent CS based on maternal respiratory illness (n=9)	Median: 38.9 (25–41.3)<br><br>(1st trimester (n=4), 2nd trimester (n=13), and 3rd trimester (n=50), postpartum (n=10)<br><br>Preterm birth (n=12)	Live birth (n=57)<br><br>Pregnant (n=20)	Yes (n=6) (mechanical ventilation (n=3); ECMO (n=1))	No (n=77)	Median: 3160 (range: 840–4350)	NICU admission (n=9)	Neonatal nasopharyngeal swab	Positive (n=4/57): on first day of life (n=3); 7 days after birth (n=1)<br><br>None of the newborns subsequently	No (n=57)

														developed respiratory symptoms	
Schnettler et al. (109)	24 Mar 2020<br><br>USA	1 case	39	Mild myotonic dystrophy (without cardiomyopathy); bicuspid aortic valve (without aortic dilation, stenosis, or regurgitation); history of a previous mild cerebrovascular accident while on combined oral contraceptives; COVID-19 pneumonia: ARDS	Caesarean	Urgent CS (Regular uterine contractions with persistent late decelerations and history of 2 previous low-transverse caesarean de-liveries)	32^{+0}	Live birth	Yes<br><br>(mechanical ventilation)	No<br><br>(patient still in hospital)	NI	NICU admission<br><br>Ventilation support (without complication was extubated on 3rd day of life)	Amniotic fluid, newborn´s nasopharyngeal swab (day 1 and day 2 - 24h apart)	Negative	No
Schoenmakers et al. (132)	Published on 9 Jun 2020<br><br>Netherlands	1 case	NI	Obesity; gestational diabetes; severe fetal distress	Caesarean	Emergency CS due to severe fetal distress	Preterm birth (3rd trimester)	Live birth	No	No	75th percentile (not specified)	NICU admission; mechanical ventilation; severe multi-organ inflammatory symptoms including coronary artery ectasia; respiratory distress syndrome; persistent pulmonary hypertension; bilateral	RT-PCR in placental tissue (maternal and fetal sides), vagina, umbilical cord blood, breastmilk, neonatal samples (blood, sputum, nasopharynx, urine, feces)	Positive: vagina; placenta (maternal and fetal sides)<br><br>Negative: nasopharynx; umbilical cord blood; breastmilk.<br><br>Serology of immunogl	NI

												intraventricular hemorrhage		obulins against SARS-CoV-2 tested negative in umbilical cord and neonatal blood.	
Sentilhes et al. (70)	Mar 1-Apr 3 2020<br><br>France	54 cases	30.6± 6.2 (age >35 =15)	Asthma (n=5); chronic hypertension (n=1); obesity (n=4); sickle-cell anemia (n=1); chronic alcohol abuse (n=1); chronic hepatitis B (n=1); nephropathy (n=1); gestational diabetes (n=4); gestational hypertensive disorders (n=2); preeclampsia (n=3)	Caesarean (n=9/21) (prelabor= 8, during labor=1)<br><br>Vaginal (n=12/21)<br><br>Miscarriage (n=1)<br><br>Pregnant (n=32)	CS related to COVID-19 infection (n=7), NI (n=2)	GA at diagnosis: 30.4±9<br><br>GA at delivery: 37.4 ± 4.7<br><br>Preterm birth (n=5) (<32 weeks=3; < 28 weeks=2)	Miscarriage (n=1)<br><br>Live births (n=21)<br><br>Pregnant (n=32)	Yes (n=5)<br><br>Invasive mechanical ventilation (n=3); ECMO (n=1); Non-invasive mechanical ventilation (n=1); oxygen support (n=13)	No<br><br>(2 still hospitalized)	2800±899 (2 SGA)	NICU admission (n=3)<br><br>Complications related to extremely or very preterm birth.	RT-PCR on neonatal throat and rectal swab samples at birth or on day 1, again on day 3 in term newborns, and also on days 7 and 14 in preterm neonates.	Negative (n=21)	No<br><br>(3 still hospitalized)
Shanes et al. (53)	8 Mar-5 May 2020<br><br>USA	1 case	26	Intrauterine fetal demise, PROM	NI	NI	16^{+0}	Intrauterine fetal demise	No	No	NA	NA	NA	NA	NA
Shanes et al. (53)	8 Mar-5 May 2020<br><br>USA	1 case	34	Gestational hypertension	NI	NI	37^{+0}	Live birth	No	No	Normal for GA	NI	Newborn´s nasopharyngeal and throat swab	Negative	No

Shanes et al. (53)	8 Mar-5 May 2020<br><br>USA	1 case	33	None	NI	NI	38^{+0}	Live birth	No	No	SGA	NI	Newborn´s nasopharyngeal and throat swab	Negative	No
Shanes et al. (53)	8 Mar-5 May 2020<br><br>USA	1 case	39	None	NI	NI	39^{+0}	Live birth	No	No	Normal for GA	NI	Newborn´s nasopharyngeal and throat swab	Negative	No
Shanes et al. (53)	8 Mar-5 May 2020<br><br>USA	1 case	31	None	NI	NI	39^{+0}	Live birth	No	No	SGA	NI	Newborn´s nasopharyngeal and throat swab	Negative	No
Shanes et al. (53)	8 Mar-5 May 2020<br><br>USA	1 case	23	Asthma	NI	NI	39^{+0}	Live birth	No	No	SGA	NI	Newborn´s nasopharyngeal and throat swab	Negative	No
Shanes et al. (53)	8 Mar-5 May 2020<br><br>USA	1 case	28	None	NI	NI	40^{+0}	Live birth	No	No	Normal for GA	NI	Newborn´s nasopharyngeal and throat swab	Negative	No
Shanes et al. (53)	8 Mar-5 May 2020<br><br>USA	1 case	26	None	NI	NI	38^{+0}	Live birth	No	No	SGA	NI	Newborn´s nasopharyngeal and throat swab	Negative	No
Shanes et al. (53)	8 Mar-5 May 2020<br><br>USA	1 case	30	None	NI	NI	38^{+0}	Live birth	No	No	Normal for GA	NI	Newborn´s nasopharyngeal and throat swab	Negative	No (Inpatient)

Shanes et al. (53)	8 Mar-5 May 2020<br><br>USA	1 case	34	None	NI	NI	40^{+0}	Live birth	No	No	SGA	NI	Neonatal nasopharyngeal and throat swab	Negative	No
Shanes et al. (53)	8 Mar-5 May 2020<br><br>USA	1 case	30	Asthma	NI	NI	34^{+0}	Live birth	No<br><br>(oxygen use)	No	Normal for GA	NI	Neonatal nasopharyngeal and throat swab	Negative	No
Shanes et al. (53)	8 Mar-5 May 2020<br><br>USA	1 case	33	None	NI	NI	37^{+0}	Live birth	No<br><br>(oxygen use)	No	Normal for GA	NI	Neonatal nasopharyngeal and throat swab	Negative	No
Shanes et al. (53)	8 Mar-5 May 2020<br><br>USA	1 case	30	None	NI	NI	39^{+0}	Live birth	No	No	Normal for GA	NI	Neonatal nasopharyngeal and throat swab	Negative	No
Shanes et al. (53)	8 Mar-5 May 2020<br><br>USA	1 case	31	None	NI	NI	40^{+0}	Live Birth	No	No	Normal for GA	NI	Neonatal nasopharyngeal and throat swab	Negative	No
Shanes et al. (53)	8 Mar-5 May 2020<br><br>USA	1 case	41	None	NI	NI	39^{+0}	Live Birth	No	No	LGA	NI	Neonatal nasopharyngeal and throat swab	Negative	No
Shanes et al. (53)	8 Mar-5 May 2020<br><br>USA	1 case	37	Gestational diabetes; cholestasis	NI	NI	37^{+0}	Live Birth	No	No	Normal for GA	NI	Neonatal nasopharyngeal and throat swab	Negative	No
Sharma et al. (110)	2 Apr 2020<br><br>India	1 case	NI	None	Caesarean	Term fetus in oblique lie position	38^{+6}	Live birth	No	No	NI	None	RT-PCR in neonatal sample (not specified) on 7 day after birth	Negative	No

Silverstein et al. (71)	Published on June 2020<br><br>USA	1 case	17	COVID-19 pneumonia	Caesarean	Clinical condition (worsened respiratory function)	36^{+6}	Live birth	Yes (mechanical ventilation)	No	3225	No	RT-PCR in neonatal sample (not specified)	Negative	No
Silverstein et al. (71)	Published on June 2020<br><br>USA	1 case	34	Obesity (BMI 40 kg/m2)	Caesarean	Clinical condition (worsened respiratory function)	34^{+1}	Live birth	Yes (mechanical ventilation)	No	2110	Admission to NICU for prematurity and monitoring	RT-PCR in neonatal sample (not specified) (day of life 1)	Negative	NI
Sun et al. (54)	Jan 2020<br><br>China	1 case	28	None	Caesarean	Confirmed or suspected maternal diagnosis of COVID-19	38^{+2}	Live Birth	NI	No	NI	None	Neonatal pharyngeal, laryngeal and throat swabs	Positive (6 days after birth)	No
Sun et al. (54)	Jan 2020<br><br>China	1 case	30	None	Caesarean	Confirmed or suspected maternal diagnosis of COVID-19	31^{+2}	Live Birth	NI	Yes	NI	NICU admission (was intubated for 4 days because of prematurity)	Neonatal pharyngeal, laryngeal and throat swabs	Negative (3 days after birth)	No
Sun et al. (54)	Jan 2020<br><br>China	1 case	29	No	Caesarean	Confirmed or suspected maternal diagnosis of COVID-19	36^{+0}	Live Birth	NI	No	NI	Asphyxia (clinically managed for 10 min without intubation); fever; COVID-19 infection based on clinical and CT scan criteria	Newborn´s pharyngeal, laryngeal and throat swabs	Negative (8 and 20 days after birth)	No

Taghizadieh et al. (111)	Published 13 May 2020<br><br>Iran	1 case	33	Acute kidney injury and acute tubular necrosis due to COVID-19 pneumonia	Caesarean	Clinical condition (dialysis 12 hours before caesarean)	34^{+1}	Live birth	NI (with mechanical ventilation)	No	NI	None	RT-PCR in neonatal sample (not specified)	Negative	No
Vallejo & Ilagan (112)<br><br>Blitz et al. (35)	Published 8 May 2020<br><br>USA	1 case	36	None	Caesarean	Reduce intraabdominal pressure	37	Live birth	Yes (with mechanical ventilation)	Yes	NI	NI	RT-PCR in neonatal sample (not specified); amniotic liquid sample	Negative	No
Vibert et al. (134)	Published on 13 May 2020<br><br>France	1 case	21	None	Pregnant	NA	GA at admission 23^{+7}	NA	Yes (alternate administration of high-flow nasal oxygen and non-invasive ventilation)	No	NA	NA	NA	NA	NA
Vintzileos et al. (154)	30 Mar-12 Apr 2020<br><br>USA	32 cases	NI	NI	NI	NI	NI	Live birth (n=32)	NI	NI	NI	NI	Neonatal nasopharyngeal swabs (n=32)	Negative (n=29)<br><br>Pending (n=3)	NI
Wang et al. (83)	2-18 Feb 2020<br><br>China	1 case	28	Fetal distress	Caesarean	Fetal distress	30^{+0}	Live birth	Yes (with mechanical ventilation)	No	1830	No	Placenta, amniotic fluid, cord blood, gastric juice at birth. Newborn´s throat swab at birth and on days 3,	Negative	No

													7 and 9 after birth. Newborn´s stool on day 3 after birth.		
Wang et al. (165)	08 Dec-01 Apr 2019-20<br><br>China	30 cases	Mean: 29.9 (Range: 26.8–33.3)	Pneumonia (n=30); hypertension (n=5); diabetes (n=2); hypothyroidism (n=1); intrahepatic cholestasis of pregnancy (n=1); PROM (n=6); Obesity (n=1)	Caesarean (n=23)<br><br>Vaginal (n=7)	Hypertension (n=1); PROM (n=1); hypothyroidism (n=1); intrahepatic cholestasis of pregnancy (n=1)	Preterm birth (n=5)	Live birth (n=31; 1 twin pregnancy)	No	No	NI	NI	RT-PCR in neonatal sample (not specified) after birth (n=31)	Negative (n=31)	No
Wen et al. (84)	22 Jan – 20 Feb 2020<br><br>China	1 case	31	None	Pregnant	NA	GA at admission 30^{+0}	NA	No	No	NA	NA	NA	NA	NA
Wu et al. (166)	31 Dec -7 Mar 2019-20<br><br>China	23 cases	Median: 29 (Range: 21-37)	Hepatitis B (n=2); hypothyroidism (n=2); pregnancy induced hypertension (n=4)	Caesarean (n=18)<br><br>Vaginal (n=2)<br><br>Voluntary termination of pregnancy (n=3)	NI	Preterm births (n=3)	Live birth (n=21; 1 twin pregnancies)	NI	No	NI	Neonatal jaundice (n=1)	RT-PCR in neonatal sample (not specified) (n=4)	Negative (n=4)	No
Wu et al. (148)	31 Jan – 9 Mar 2020<br><br>China	1 case	29	Fetal Distress	Caesarean	Fetal Distress	35^{+6}	Live birth	No	No	2830	Neonatal pneumonia	Neonatal throat and anal swabs (1st and 3rd days after birth);	Negative	No

													vaginal secretions sample; breastmilk		
Wu et al. (148)	31 Jan – 9 Mar 2020<br><br>China	1 case	26	None	Caesarean	COVID-19 potential mother-to-child transmission	35^{+5}	Live Birth	No	No	2300	Neonatal pneumonia	Neonatal throat and anal swabs (1st and 3rd days after birth); vaginal secretions sample	Negative	No
Wu et al. (148)	31 Jan – 9 Mar 2020<br><br>China	1 case	29	None	Caesarean	COVID-19 potential mother-to-child transmission	38^{+4}	Live birth	No	No	2650	Neonatal asphyxia	Neonatal throat and anal swabs (1st and 3rd days after birth); vaginal secretions sample	Negative	No
Wu et al. (148)	31 Jan – 9 Mar 2020<br><br>China	1 case	27	PROM	Vaginal	NA	38+2	Live birth	No	No	3910	NI	Neonatal throat swab, vaginal secretions samples; breastmilk	Negative<br><br>Breastmilk 1st after delivery: positive<br>Breastmilk 3rd after delivery: negative	No
Wu et al. (148)	31 Jan – 9 Mar 2020<br><br>China	1 case	31	None	Pregnant	NA	GA at admission: 32^{+4}	NA	No	No	NA	NA	NA	NA	NA

Wu et al. (148)	31 Jan – 9 Mar 2020 China	1 case	40	None	Pregnant	NA	GA at admission: 8^{+5}	NA	No	No	NA	NA	NA	NA	NA
Wu et al. (148)	31 Jan – 9 Mar 2020 China	1 case	36	None	Pregnant	NA	GA at admission: 16^{+1}	NA	No	No	NA	NA	NA	NA	NA
Wu et al. (148)	31 Jan – 9 Mar 2020 China	1 case	28	None	Pregnant	NA	GA at admission: 6^{+3}	NA	No	No	NA	NA	NA	NA	NA
Wu et al. (148)	31 Jan – 9 Mar 2020 China	1 case	27	None	Pregnant	NA	GA at admission: 11^{+2}	NA	No	No	NA	NA	NA	NA	NA
Wu et al. (148)	31 Jan – 9 Mar 2020 China	1 case	29	Chronic nephritis	Pregnant	NA	GA at admission: 17^{+0}	NA	No	No	NA	NA	NA	NA	NA
Wu et al. (148)	31 Jan – 9 Mar 2020 China	1 case	32	None	Chemical pregnancy	NA	GA at admission: 5^{+1}	Chemical pregnancy	No	No	NA	NA	NI	NI	NA
Wu et al. (148)	31 Jan – 9 Mar 2020 China	1 case	35	None	Pregnant	NA	GA at admission: 9^{+3}	NA	No	No	NA	NA	NA	NA	NA
Wu et al. (72)	23 Jan -10 Feb 2020 China	1 case	28	NI	Caesarean	History of C-section	GA at admission 39^{+0}	NI	No	No	NI	NI	NI	NI	NI

Wu et al. (72)	23 Jan -10 Feb 2020 China	1 case	31	PROM	Caesarean	PROM	GA at admission 38^{+1}	NI	No	No	NI	NI	NI	NI	NI
Wu et al. (72)	23 Jan -10 Feb 2020 China	1 case	30	Preeclampsia	Caesarean	Preeclampsia	GA at admission 39^{+1}	NI	No	No	NI	NI	NI	NI	NI
Wu et al. (72)	23 Jan -10 Feb 2020 China	1 case	30	PROM	Caesarean	PROM	GA at admission 36^{+4}	NI	No	No	NI	NI	NI	NI	NI
Wu et al. (72)	23 Jan -10 Feb 2020 China	1 case	30	NI	Vaginal	NA	GA at admission 37^{+6}	NI	No	No	NI	NI	NI	NI	NI
Wu et al. (72)	23 Jan -10 Feb 2020 China	1 case	26	Fetal distress	Caesarean	Fetal distress	GA at admission 40^{+3}	NI	No	No	NI	NI	NI	NI	NI
Wu et al. (72)	23 Jan -10 Feb 2020 China	1 case	29	NI	Vaginal	NA	GA at hospital admission 40^{+4}	NI	No	No	NI	NI	NI	NI	NI
Wu et al. (72)	23 Jan -10 Feb 2020 China	1 case	35	NI	Caesarean	History of C-section	GA at hospital admission 33^{+6}	NI	No	No	NI	NI	NI	NI	NI
Xia et al. (85)	20 Jan 2020 China	1 case	27	Reduced fetal movement, little amniotic fluid, possible intrauterine distress	Caesarean	Possible fetal distress; COVID-19 pneumonia	37^{+2}	Live birth	NI	No	3100	NI	Neonatal oropharyngeal swab (day 3 and 5 after birth)	Negative	No

Xiong et al. (113)	29 Jan – 10 Mar 2020<br><br>China	1 case	25	PROM	Vaginal	NA	38^{+4}	Live birth	No	No	3070	None	RT-PCR of: amniotic fluid, neonatal throat swab, neonatal rectal swab; breastmilk<br><br>Immunohistochemically analysis for N protein of SARS-CoV-2 detection in the placenta<br><br>Neonatal IgG and IgM antibodies to SARS-CoV-2	Negative: amniotic fluid, neonatal throat swabs, breastmilk, placenta<br><br>Non-reactive for neonatal IgG and IgM antibodies to SARS-CoV-2	No
Xu et al. (56)	21 Jan – 09 Feb 2020<br><br>China	1 case	34	COVID-19 pneumonia	Caesarean	Abdominal pain	GA at admission 38^{+6}	Live birth	No	No	3050	Scattered skin rashes	Neonatal throat swab (8 days after birth)	Negative	No
Xu et al. (56)	21 Jan – 09 Feb 2020<br><br>China	1 case	25	COVID-19 pneumonia	Caesarean	COVID-19 pneumonia	34^{+4}	Live birth	No	No	2580	None	Neonatal throat swab (3 days after birth)	Negative	No

Xu et al. (56)	21 Jan – 09 Feb 2020<br><br>China	1 case	23	COVID-19 pneumonia; polyhydramnios; placenta previa	Caesarean	COVID-19 pneumonia	GA at admission 37^{+3}	Live birth	No	No	3760	None	Neonatal throat swab (8 days after birth)	Negative	No
Xu et al. (56)	21 Jan – 09 Feb 2020<br><br>China	1 case	34	COVID-19 pneumonia	Caesarean	COVID-19 pneumonia	GA at admission 36^{+4}	Live birth	No	No	2450	None	Neonatal throat swab (1 day after birth)	Negative	No
Xu et al. (56)	21 Jan – 09 Feb 2020<br><br>China	1 case	28	COVID-19 pneumonia; colporrhagia	Vaginal	NA	GA at admission 37^{+0}	Live birth	No	No	3120	None	Neonatal throat swab	Negative	No
Yan et al. (57)<br><br>Yang et al. (58)<br><br>Chen et al. (11)<br><br>Liu et al. (76)<br><br>Zhang et al. (87)<br><br>Zhang et al. (31)	20 Jan-24 Mar 2020<br><br>China	116 cases	Mean: 30.8 (±3.8) (Range: 24-41)	COVID-19 pneumonia (n=116)<br><br>Gestational diabetes (n=9), Hypertensive disorders (n=5), Preeclampsia (n=4), PPROM (n=6), hypothyroidism (n=1), fetal distress (n=9)	Caesarean (n=85)<br><br>Vaginal (n=14)<br><br>Spontaneous abortion at 5^{+2} weeks (n=1)<br><br>Pregnant (n=16)	COVID-19 pneumonia (n=33), placenta previa (n=3), previous caesarean delivery (n=16), fetal distress (n=9), preeclampsia (n=4), failure to progress (n=5), abnormal fetal growth (n=2),	Median: 38.4 (IQR:37.3, 39.4)<br><br>Preterm birth (n= 21)	Live birth (n=100; 1 twin pregnancy)	Yes (n=8) (invasive mechanical ventilation (n=2); ECMO (n=1))	No	Mean: 3108±526	Severe neonatal asphyxia (n=1)<br><br>NICU admission (n=47)	Neonatal pharyngeal samples (n=86); amniotic fluid and cord blood samples (n=10); breastmilk samples (n=12); maternal vaginal samples (n=6)	Negative	Yes (n=1)

Chen et al. (19)						twin pregnancy (n=1)									
Yang et al. (149)	20 Jan – 19 Mar 2020<br><br>China	24 cases	Mean 29.91± 3.61 (Range: 22-39)	Hepatitis B (n=1), schistosomiasis (n=1), Gestational diabetes (n=2), Dysfunction of blood coagulation (n=2), Hypothyroid (n=2), Gestational hypertension (n=2), severe preeclampsia (n=1), Hypoproteinemia (n=1), abnormal umbilical cord (n=4), intrauterine distress (n=3), abnormal amniotic fluid (n=1), PROM (n=2), COVID-19 pneumonia (n=23)	Caesarean (n=16)<br><br>Vaginal (n=4)<br><br>Voluntarily chosen induced abortion due to COVID-19 (n=4)	NI	Median: 38.0 (IQR: 37.3-39.1)	Live birth (n=20)	NI	No	Mean: 3,290 ±297	None	Neonatal throat swabs (n=19)	Negative (n=19)	No
Yang et al. (149)	20 Jan – 19 Mar 2020<br><br>China	1 case	28	Viral hepatitis; Dysfunction of blood coagulation; COVID-19 pneumonia	Vaginal	NI	38^{+5}	Live birth	NI	No	3370	None	Neonatal throat swabs	Negative	No
Yang et al. (149)	20 Jan – 19 Mar 2020<br><br>China	1 case	26	COVID-19 pneumonia	Caesarean	NI	37^{+2}	Live birth (n=2; twin pregnancy)	NI	No	T1: 2350 T2: 2620	None	Neonatal throat swabs	Negative	No

Yang et al. (149)	20 Jan – 19 Mar 2020<br><br>China	1 case	39	PPROM, Gestational Diabetes; COVID-19 pneumonia	Caesarean	NI	30^{+6}	Live birth	NI	No	1500	severe neonatal asphyxia	Neonatal throat swabs; neonatal blood serologic testing	Negative<br><br>Reactive IgG and IgM	No
Yang et al. (171)	20 Jan – 05 Mar 2020<br><br>China	13 cases	Mean 30.2 ± 2.3	NI	Caesarean (n=9)<br><br>Vaginal (n=4)	NI	Mean: 38.2 ± 2.3<br><br>Preterm birth (n=2)	Live birth (n=14, 1 twin pregnancy)	NI	No	Mean: 3063.2 ± 536.4	Neonatal respiratory distress syndrome after birth (n=2); NICU admission (n=14)	NI	NI	No
Yang et al. (58)	20 -29 Jan 2020<br><br>China	1 case	NI	NI	Caesarean	COVID-19	36^{+0}	Live birth	NI	No	3800	NICU admission (respiratory support – nCPAP)	Umbilical cord blood, amniotic fluid and neonatal pharyngeal swab at birth<br><br>Neonatal Pharyngeal swabs 2−5 days after birth	Negative	No
Yang et al. (58)	20 -29 Jan 2020<br><br>China	1 case	NI	NI	Caesarean	COVID-19	36^{+2}	Live birth	NI	No	2620	None	No	NA	No
Yassa et al. (73)	Published on June 2020<br><br>Turkey	1 case	32	NI	Caesarean	Prolonged pre-labor rupture of membranes	39	Live birth	No	No	3070	No	Neonatal nasopharyngeal and throat swabs	Negative	No

Yassa et al. (73)	Published on June 2020<br><br>Turkey	1 case	32	NI	Pregnant	NA	GA at admission 27 weeks	NA	No	No	NA	NA	NA	NA	NA
Yassa et al. (73)	Published on June 2020<br><br>Turkey	1 case	33	NI	Pregnant	NA	GA at admission 20 weeks	NA	Yes	No	NA	NA	NA	NA	NA
Yassa et al. (73)	Published on June 2020<br><br>Turkey	1 case	19	NI	Pregnant	NA	GA at admission 9 weeks	NA	No	No	NA	NA	NA	NA	NA
Yassa et al. (73)	Published on June 2020<br><br>Turkey	1 case	41	NI	Pregnant	NA	GA at admission 17 weeks	NA	No	No	NA	NA	NA	NA	NA
Yassa et al. (73)	Published on June 2020<br><br>Turkey	1 case	40	Vaginal bleeding	Miscarria ge	NA	7	Miscarriage	No	No	NA	NA	NA	NA	NA
Yassa et al. (73)	Published on June 2020<br><br>Turkey	1 case	23	NI	Pregnant	NA	GA at admission 10 weeks	NA	No	No	NA	NA	NA	NA	NA
Yassa et al. (73)	Published on June 2020<br><br>Turkey	1 case	40	NI	Caesarean	Maternal request	38	NI	No	No	NI	NI	NI	NI	NI

Yilmaz et al. (135)	May 2020<br><br>Turkey	1 case	25	NI	Caesarean	NI	38	Live birth	No	No	2900	No	RT-PCR neonatal samples	Negative	No
Yin et al. (150)	28 Jan- 28 Feb 2020<br><br>China	31 cases	Mean: 31.0±4.3	NI	Caesarean (n=13)<br><br>Vaginal (n=4)<br><br>Uterine curettage (personal choice) in 1st trimester (n=3)<br><br>Pregnant (n=11)	NI	Preterm births (≥35 weeks) (n=5)<br><br>Term births (n=12)	Live birth (n=17)	NI	No	Preterm infants: 2580 (range 2450-3130)<br><br>Term infants: 3035 (range: 2830-4100)	None	Breastmilk (n=14), amniotic fluid (n=2), placenta (n=2), neonatal throat swabs (n=17) and neonatal anal swabs (n=5)	Negative	No
Yu et al. (114)	28 Jan-7 Feb 2020<br><br>China	1 case	35	NI	Vaginal	NA	34^{+0}	Live birth	Yes after delivery (with tracheal intubation – severe ARDS)	No	NI	None	Neonatal throat swab	Negative	No
Yu et al. (59)	Jan-Feb 2020<br><br>China	1 case	34	NI	Pregnant	NA	GA at admission 8^{+2}weeks	NA	No	No	NA	NA	Maternal amniotic fluid<br><br>Serologic tests in amniotic fluid	Negative<br><br>Non-reactive for SARS-CoV-2 IgM and IgG	NA

Yu et al. (59)	Jan – Feb 2020<br><br>China	1 case	27	NI	Pregnant	NA	GA at admission 10^{+1}weeks	NA	No	No	NA	NA	Maternal amniotic fluid<br><br>Serologic tests in amniotic fluid	Negative<br><br>Non-reactive for SARS-CoV-2 IgM and IgG	NA
Zamaniyan et al. (113)	Mar 2020<br><br>Iran	1 case	22	Hypothyroidism; ARDS	Caesarean	COVID-19 disease; low lymphocytes counts and unfavorable cervix for induction	GA at admission 32^{+0}<br><br>Preterm birth	Live birth	Yes<br>(With peritoneal dialysis due to ARDS, intubation)	Yes	2350	Fever	umbilical cord blood, maternal vaginal secretion, neonatal nasal and throat swabs, amniotic fluid<br><br>Neonatal swabs one week after birth	Negative (umbilical cord blood; vaginal secretions; neonatal swabs<br><br>Positive (amniotic fluid; neonatal swabs 1 week after birth)	No
Zambrano et al. (86)	9 Mar - 19 Mar 2020<br><br>Honduras	1 case	41	Gestational hypertension and hypothyroidism	Vaginal	NA	32^{+0}	Live birth	No	No	1500	Dysplastic and multicystic right kidney	Newborn´s nasopharyngeal swab and blood sample	Negative	No
Zeng et al. (30)	16 Feb - 6 Mar 2020<br><br>China	6 cases	SI	NI	Caesarean (n=6)	NI	NI	Live births	NI	No	SI	NI	Neonatal blood and throat swab.	Negative RT-PCR test results in blood and throat swab (n=6); High IgG and IgM concentrat	NI

															ions (n=2); High IgG and normal IgM (n=3)	
Zeng et al. (151)	Jan - Feb 2020<br>China	1 case	NI	None	Caesarean	Meconium-stained amniotic fluid and confirmed COVID-19 pneumonia	40^{+0}	Live birth	No	No	3250	Lethargy, Fever. Radiologic diagnosis of pneumonia	Neonatal nasopharyngeal and anal swab (day 2, 4 and 6 after birth)	Positive on day 2 and 4 and negative on day 6 (oral and anal swabs)	No
Zeng et al. (151)	Jan - Feb 2020<br>China	1 case	NI	None	Caesarean	COVID-19 Pneumonia	40^{+4}	Live birth	No	No	3360	Lethargy, vomiting, and fever. Radiologic diagnosis of pneumonia	Neonatal nasopharyngeal and anal swab (day 2, 4 and 6 after birth)	Positive on day 2 and 4 and negative on day 6 (oral and anal swabs)	No
Zeng et al. (151)	Jan - Feb 2020<br>China	1 case	NI	PROM; Fetal distress	Caesarean	Fetal distress, COVID-19 pneumonia	31^{+2}	Live birth	No	No	1580	Asphyxia, Pneumonia, Respiratory distress syndrome, shortness of breath, cyanosis. Mechanical ventilation	Neonatal nasopharyngeal and anal swab (day 2, 4 and 7 after birth)	Positive on day 2 and 4 and negative on day 7 (oral and anal swabs)	No
Zeng et al. (151)	Jan - Feb 2020<br>China	30 cases	NI	PROM (n=2)	Vaginal (n=7);<br>Caesarean (n=23)	NI	Preterm births (n=3)	Live birth (n=30)	No	No	SGA (n=2)	Asphyxia (n=1); Respiratory distress syndrome (n=3); shortness of breath (n=3); cyanosis (n=2); feeding	RT-PCR in neonatal sample (not specified)	Negatives (n=30)	No

												intolerance (n=2)			
Zeng et al. (155)	Until Feb 16 2020<br><br>China	16 cases	Mean: 31 ± 3.84<br><br>(Range: 25–40)	PROM (n=3), cardiac disease in pregnancy (n=2), hypothyroidism (n=2), polyhydramnios (n=1), thalassemia (n=1), intrauterine growth restriction (n=1), fetal macrosomia (n=1)	Caesarean (n=12)<br><br>Vaginal (n=4)	NI	Median GA: 37^{+5}<br><br>(Range: 34^{+3}-41^{+1})<br><br>Preterm birth (n=3)	Live birth (n=16)	No	No	Mean: 3175.37 ± 478.58<br><br>(Range: 2450–4100)	NI	Neonate RT-PCR	Negative (n=16)	No
Zeng et al. (167)	20 Jan – 08 Feb 2020<br><br>China	3 cases	NI	ARDS (n=1)	Caesarean (n=2)<br><br>Pregnant (n=1)	Fetal heart rate instability and possible fetal respiratory distress (n=2)	Preterm birth (30^{+0}) (n=1)<br><br>Term birth (37^{+0}) (n=1)	Live birth (n=2)	Yes (invasive mechanical ventilation and ECMO treatment) (n=1)	Yes (n=1)	NI	Pneumonia (n=1)	Neonatal throat swab samples (n=2)	Negative (n=2)	No
Zhang et al. (168)	8 Dec - 13 Mar 2020<br><br>China	4 cases	NI	NI	Caesarean (n=4)	NI	Term (n=2)<br><br>Preterm (n=2)	Live birth (n=4)	No	No	NI	None	Neonatal nasopharyngeal (n=2); neonatal anal swabs (n=2)	Positive (n=4)	No (n=4)
Zhu et al. (74)	1 Feb-25 Mar 2020<br><br>China	1 case	29	NI	Caesarean	NI	35^{+5}	Live birth	No	No	NI	None	Vaginal secretion, breastmilk	Negative	No
Zhu et al. (74)	1 Feb-25 Mar 2020<br><br>China	1 case	29	NI	Caesarean	NI	35^{+0}	Live birth	No	No	NI	None	Vaginal secretion, breastmilk	Negative	No
Zhu et al. (74)	1 Feb-25 Mar 2020	1 case	34	COVID-19 pneumonia	Caesarean	NI	40^{+0}	Live birth	No	No	NI	None	Breastmilk	Positive (after delivery	No

	China													for two and three days)	
Zhu et al. (74)	1 Feb-25 Mar 2020<br><br>China	1 case	27	NI	Vaginal	NA	38^{+2}	Live birth	No	No	NI	None	Vaginal secretion, breastmilk	Negative	No
Zhu et al. (74)	1 Feb-25 Mar 2020<br><br>China	1 case	32	NI	Caesarean	NI	40^{+1}	Live birth	No	No	NI	None	Breastmil k	Negative	No
Tang et al. (133)	Apr 2020<br><br>Netherlan ds	1 case	NI	None	Caesarean	Clinical condition	41	Live birth	No	No	NI	NI	NI	NI	No

Abbreviations: ARDS – Acute Respiratory Distress Syndrome; ART - Assisted Reproductive Technology; CS – Caesarean section; ECMO – Extracorporeal Membrane Oxygenation; GA – Gestational Age; MODS - Multiple Organ Dysfunction Syndrome; NA – Not Applicable; NI – No Information; NICU – Neonatal Intensive Care Unit; PROM – Premature Rupture of Membranes; RT-PCR – Real-time reverse Transcriptase–Polymerase Chain Reaction; SCIM - Septic Induced Ischemic Cardiomyopathy; SGA – Small for Gestational Age.
